# NanoDam identifies Homeobrain (ARX) and Scarecrow (NKX2.1) as conserved temporal factors in the *Drosophila* central brain and visual system

**DOI:** 10.1016/j.devcel.2022.04.008

**Published:** 2022-05-09

**Authors:** Jocelyn L.Y. Tang, Anna E. Hakes, Robert Krautz, Takumi Suzuki, Esteban G. Contreras, Paul M. Fox, Andrea H. Brand

**Affiliations:** 1 Gurdon Institute and Department of Physiology, Development and Neuroscience, University of Cambridge, Tennis Court Road, Cambridge CB2 1QN, UK

**Keywords:** NanoDam, neural stem cells, temporal patterning, neural development, ARX, NKX2.1

## Abstract

Temporal patterning of neural progenitors is an evolutionarily conserved strategy for generating neuronal diversity. Type II neural stem cells in the *Drosophila* central brain produce transit-amplifying intermediate neural progenitors (INPs) that exhibit temporal patterning. However, the known temporal factors cannot account for the neuronal diversity in the adult brain. To search for missing factors, we developed NanoDam, which enables rapid genome-wide profiling of endogenously tagged proteins *in vivo* with a single genetic cross. Mapping the targets of known temporal transcription factors with NanoDam revealed that Homeobrain and Scarecrow (ARX and NKX2.1 orthologs) are also temporal factors. We show that Homeobrain and Scarecrow define middle-aged and late INP temporal windows and play a role in cellular longevity. Strikingly, Homeobrain and Scarecrow have conserved functions as temporal factors in the developing visual system. NanoDam enables rapid cell-type-specific genome-wide profiling with temporal resolution and is easily adapted for use in higher organisms.

## Highlights

• NanoDam: rapid genome-wide profiling of endogenously tagged chromatin-binding proteins
• NanoDam allows precise spatial and temporal profiling *in vivo* without cell isolation
• Scro and Hbn are temporal factors in both the CB and the visual system
• Regulatory interactions of Scro (NKX2.1) and Hbn (ARX) are also conserved

## Graphical abstract

**Figure:**
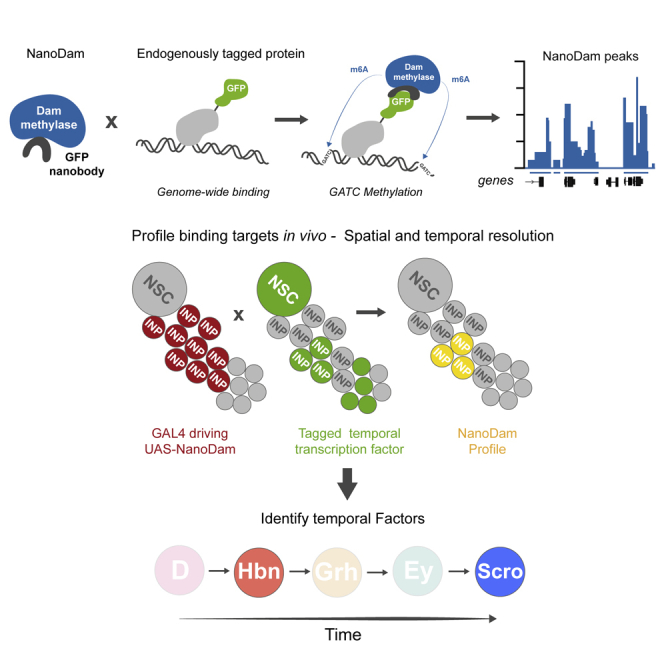

## Teaser

In NanoDam, a nanobody targets Dam methylase to endogenously tagged DNA-binding proteins for genome-wide profiling *in vivo*. By combining results from NanoDam and scRNA-seq, Tang et al. identify conserved temporal transcription factors acting in neural progenitors to regulate neuronal patterning in the *Drosophila* central brain and optic lobe.

## Introduction

The nervous system is generated by a relatively small number of neural stem cells (NSCs) and progenitors that are patterned both spatially and temporally (Holguera and Desplan, 2018). Spatial patterning confers differences between populations of NSCs, whereas changes in gene expression over time direct the birth order and subtype identity of neuronal progeny. Temporal transcription factor (TF) cascades determine neuronal birth order in the *Drosophila* embryonic central nervous system (CNS), the larval central brain (CB), and optic lobe (OL) (Doe, 2017). In the CB, type II NSCs generate transit-amplifying intermediate neural progenitors (INPs), which divide asymmetrically to self-renew and generate daughter cells (ganglion mother cells or GMCs) in a manner analogous to human outer radial glial (oRG) cells (Bello et al., 2008; Boone and Doe, 2008; Bowman et al., 2008; Hansen et al., 2010). GMCs in turn undergo a terminal cell division, generating neurons or glial cells that contribute to the adult central complex (Bayraktar and Doe, 2013; Bayraktar et al., 2010; Izergina et al., 2009; Viktorin et al., 2011). The sequential divisions of INPs increase the quantity of neurons, which in turn creates a platform for generating wider neuronal diversity: 8 type II NSCs in each brain lobe give rise to the adult central complex, composed of at least 60 different neuronal subtypes (Young and Armstrong, 2010). The tight control of progenitor temporal identity is crucial for the production of neuronal subtypes at the appropriate time and in the correct numbers.

**Figure 1:**
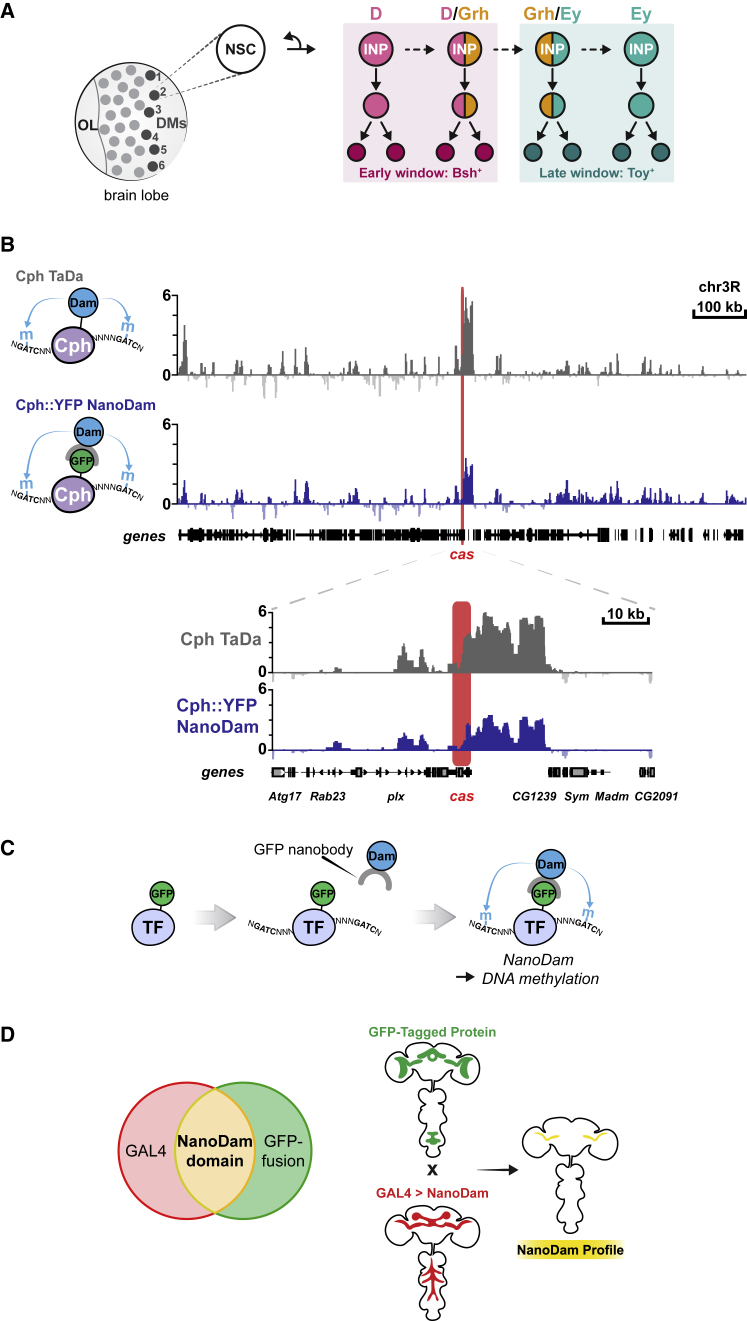
NanoDam profiles the genome-wide binding sites of GFP-tagged transcription factors in their endogenous temporal windows (A) In the *Drosophila* larval CNS, the 6 dorsal-medial (DM) type II NSCs in each brain lobe generate INPs that sequentially express D, Grh, and Ey. The expression of D/Grh versus Grh/Ey defines early and late INP temporal windows and contributes to neuronal diversity. (B) Comparison of Cph TaDa and NanoDam (using Cph::YFP) binding across a region of chromosome 3r. Note that *cas* (highlighted in red), which we previously identified as a target of Cph using TaDa, is detected with both techniques. Binding intensities are shown as log_2_-fold enrichment of Cph TaDa over TaDa only and Cph::YFP NanoDam over NanoDam only. (C)To create NanoDam, a GFP nanobody was fused to the Dam protein. The nanobody can bind to the GFP-tagged transcription factor (TF) and recruits Dam to the TF binding sites, resulting in genome-wide GATC methylation. (D) NanoDam allows for increased spatial resolution of profiling.

The INPs produced by the 6 dorsal-medial type II lineages (DM1–6) sequentially express the temporal TFs Dichaete (D, a member of the Sox family), Grainyhead (Grh, a Grh/CP2 family TF) and Eyeless (Ey, a homolog of Pax6) (Figure 1A; Bayraktar and Doe, 2013). These temporal factors were discovered initially by screening type II lineages for restricted expression of neural TFs, using 60 different antisera (Bayraktar and Doe, 2013). This non-exhaustive approach was able to find a fraction of the theoretically necessary temporal factors, leaving the true extent of temporal regulation and the identity of missing temporal factors open. Furthermore, the cross-regulatory interactions predicted in a temporal cascade, in which each temporal TF activates expression of the next temporal factor and represses expression of the temporal factor preceding it, are not fulfilled solely by D, Grh, and Ey (Baumgardt et al., 2009; Brody and Odenwald, 2000; Cleary and Doe, 2006; Grosskortenhaus et al., 2005, 2006; Isshiki et al., 2001; Kambadur et al., 1998; Novotny et al., 2002; Pearson and Doe, 2003; Tran and Doe, 2008; Li et al., 2013).

Three further factors contribute to INP temporal progression, but they are expressed broadly rather than in discrete temporal windows: Osa, a SWI/SNF chromatin remodeling complex subunit; and two further TFs, Odd-paired (Opa) (Abdusselamoglu et al., 2019) and Hamlet (Ham) (Eroglu et al., 2014). Therefore, there must exist other TFs that are expressed in defined temporal windows and that exhibit the regulatory interactions expected in a temporal cascade. We postulated that other temporal factors remain to be identified.

Given the feedforward and feedback transcriptional regulation previously observed in temporal transcription cascades, we surmised that previously unidentified temporal factors would be among the transcriptional targets of D, Grh, or Ey. Therefore, we devised NanoDam to identify the genome-wide targets of TFs within their normal expression windows *in vivo* without cell isolation, cross-linking, or immunoprecipitation. Temporal factors are expressed transiently in a small pool of rapidly dividing progenitor cells. NanoDam provides a simple, streamlined approach to obtain genome-wide binding profiles in a cell-type-specific and temporally restricted manner.

Using NanoDam, we identified the transcriptional targets of D, Grh, and Ey in INPs and, by performing single-cell RNA sequencing (scRNA-seq), determined which of the directly bound loci were activated or repressed. Next, we assessed which of the target loci encoded TFs and whether these were expressed in restricted temporal windows within INPs. We surveyed where in the INP transcriptional cascade these factors acted and ascertained whether they cross-regulate the expression of other temporal TF genes, as expected for temporal factors. Finally, we showed that Hbn and Scro act as temporal factors, playing similar roles and exhibiting the same cross-regulatory interactions, in the temporal cascade of the developing visual system. This is particularly striking as the INPs and the NSCs of the developing OL have different cells of origin, and yet the mechanism they use to generate neuronal diversity is conserved.

## Design

To identify further temporal TFs, we set out to profile the genome-wide binding targets of D, Grh, and Ey, within their normal temporal windows. We created NanoDam, which capitalizes on our targeted DamID technique (TaDa), in which the DNA- or chromatin-binding protein of interest is fused to an *E. coli* Dam methylase (Marshall and Brand, 2015; Marshall et al., 2016; Southall et al., 2013). As in DamID, when the Dam-fusion protein interacts with the genome, it methylates adenine within the sequence GATC (van Steensel and Henikoff, 2000; van Steensel et al., 2001). Endogenous adenine methylation is extremely rare in eukaryotes (Koziol et al., 2016; Wu et al., 2016; Zhang et al., 2015), such that the genomic targets of the Dam-fusion protein can be identified readily by mapping adenine methylation in the genome (see for example, Marshall et al., 2016). TaDa enables cell-type-specific genome-wide profiling *in vivo*, using the GAL4 system (Brand and Perrimon, 1993), while avoiding the potential toxicity resulting from the expression of high levels of the Dam methylase (Southall et al., 2013). For each TaDa experiment, however, a transgene must first be generated encoding the Dam methylase fused to a candidate protein. The transgene is then ectopically expressed, albeit at very low levels, driven by a cell-type-specific GAL4 driver (Southall et al., 2013).

In designing NanoDam, we sought to benefit from all of the advantages of TaDa while, first, bypassing the need to generate recombinant transgenes and, second, assessing genome-wide binding only when and where the DNA- or chromatin-binding protein is normally expressed. NanoDam recruits the Dam methylase to endogenously tagged proteins, using nanobodies as targeting agents. Nanobodies are recombinant antibody fragments derived from the variable region of heavy-chain antibodies present in camelid species (Muyldermans, 2001). We fused the Dam methylase to a nanobody recognizing GFP in order to direct the Dam methylase-nanobody fusion to any GFP-tagged protein and enable genome-wide profiling of the tagged protein (Figure 1C).

We fused nanobody vhhGFP4—which recognizes GFP and a number of its variants, including eGFP, YFP, CFP, BFP, and Venus (Caussinus et al., 2011)—to the C terminus of Dam methylase (Figure S1A) and used TaDa (Southall et al., 2013) to drive low levels of tissue-specific expression. With TaDa, a bicistronic message is transcribed: a primary open reading frame 1 (ORF1; here mCherry) followed by two TAA stop codons; and a single-nucleotide frameshift upstream of a secondary ORF, in this case the coding sequence of the Dam-nanobody fusion protein (ORF2). Translation of this bicistronic message results in expression of ORF1 as well as extremely low levels of expression of the Dam-nanobody fusion protein (ORF2) due to rare ribosomal re-entry and translational re-initiation.

## Results

### NanoDam accurately identifies genome-wide chromatin-binding sites

To assess the efficacy of NanoDam, we profiled binding of the TF CG9650, the *Drosophila* homolog of CTIP^1/2^/Bcl11^a/b^, which we call Chronophage (Cph). The *cph* locus had been tagged by insertion of a YFP protein trap (Cph::YFP), resulting in Cph-YFP expression from its own promoter in the embryonic CNS (Lowe et al., 2014). We drove expression of NanoDam in embryonic NSCs with *worniu*-GAL4 and compared our results from NanoDam with those we obtained using TaDa. NanoDam for Cph::YFP and TaDa for Cph (2 replicates of each), were performed under the same conditions (see STAR Methods).

NanoDam accurately reproduced the binding profiles obtained with TaDa genome-wide and at individual loci, as exemplified at the *castor* locus (Figure 1B). We performed genome-wide correlation analyses to compare and contrast the binding profiles produced by NanoDam and TaDa. The binding profiles of both showed high correlation between individual replicates of Dam only or NanoDam only, and Cph TaDa or Cph::YFP NanoDam (Figure S1B). In addition, we assessed the fraction of unique reads of the individual library and cumulative fraction of reads across all genomic bins as a readout of library complexity (Figure S1C). These quantifications showed that the NanoDam libraries, at the same sequencing depth, have a higher signal-to-noise ratio as well as more significant genome-wide Dam methylation compared with TaDa.

Next, we assessed the genome-wide binding intensities of Cph TaDa and Cph::YFP NanoDam normalized over their respective controls. Normalized Cph NanoDam binding profiles correlated highly with one another and less with their TaDa counterparts, in line with the library complexity results (Figure S1E). When control samples were compared with one another, they showed random signal profiles with low signal intensities (Figure S1D) and did not correlate genome-wide with other samples (Figure S1E).

To compare identified sets of peaks across both replicates and methods, we used receiver operating characteristic (ROC)-like curves to measure peak recovery between individual replicates and references (one reference used for each, NanoDam or TaDa; Figure S1F). Combined, the two plots indicate that TaDa peaks not only overlap with other TaDa replicates, but also with NanoDam-identified peaks. On the other hand, NanoDam peaks are recovered less well by TaDa, demonstrating that TaDa peaks are a subset of NanoDam peaks. Finally, peak enrichment analysis of data obtained from both methods is consistent with the genome-wide comparison as NanoDam signal is not only stronger on peak sets derived from NanoDam replicates alone, but it is also a union of NanoDam and TaDa peaks (Figure S1G). Moreover, the NanoDam signal almost completely recapitulates TaDa binding intensities (Figure S1G). Taken together with the ROC-like curve analyses (Figure S1F), our data show that NanoDam identifies Cph binding sites with a higher sensitivity than TaDa.

By restricting expression of the NanoDam construct using different GAL4 drivers, the genome-wide binding pattern of any tagged factor can be assessed in a defined subset of its endogenous expression pattern. Specific genomic DNA methylation occurs in cells that express both the NanoDam construct (under the control of GAL4) and the endogenously tagged protein (under the control of its own regulatory elements) (Figure 1D). This is particularly important when profiling proteins that are expressed only in a subset of a cells within a lineage, a fact we sought to exploit for identifying temporal TFs. Therefore, we were confident that NanoDam could be used to profile the genome-wide occupancy of the temporal TFs D, Grh, and Ey.

### NanoDam reveals combinatorial binding patterns of the INP temporal factors

**Figure 2:**
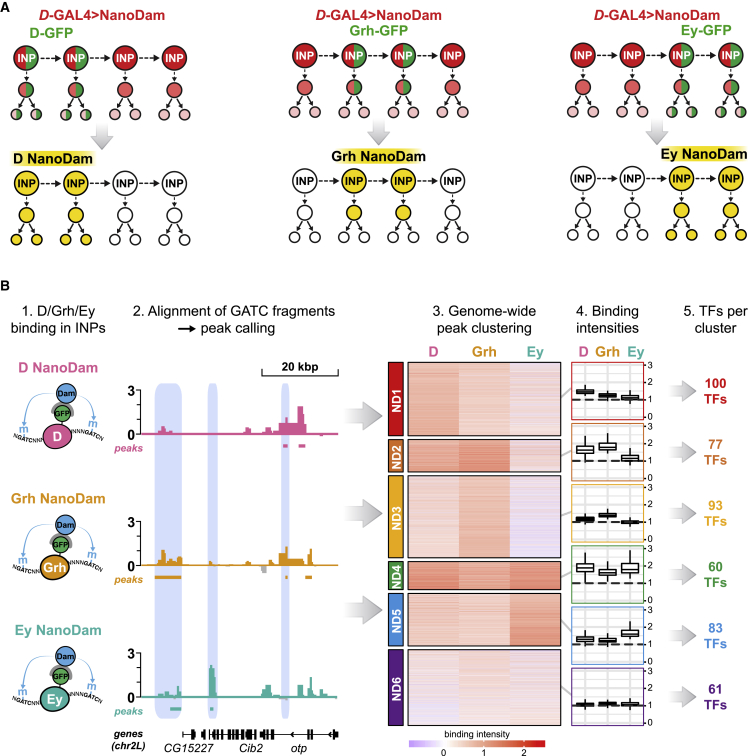
NanoDam for INP temporal factors D, Grh and Ey (A) The experimental setup for D, Grh, and Ey NanoDam in INPs. *D*-GAL4 drives UAS-*NanoDam* (red) in all INPs of DM1–6 during late third-instar larval stage. D-GFP, Grh-GFP, and Ey-GFP (green) are expressed in a subset of INPs and progeny, restricting NanoDam binding to their respective temporal windows (yellow). Note that *D*-GAL4 expression extends beyond the endogenous temporal window of D. (B) NanoDam-derived binding intensities for D, Grh, and Ey were aggregated for highly significant peaks identified by comparison with the *w*^*1118*^ control. Unsupervised clustering of the peaks according to these intensities identified 6 distinct combinations of D/Grh/Ey binding, denoted as ND1–6. Binding intensities are shown as *Z* scores for individual peaks in the heatmaps. Average binding intensities across all peaks per NanoDam cluster are represented as boxplots. Binding intensities are shown as log_2_-fold enrichment TF-GFP NanoDam over NanoDam only. 4 D-GFP replicates, 5 Grh-GFP replicates, and 4 Ey-GFP replicates were normalized individually to all control replicates (8 *w*^*1118*^ replicates).

We expressed NanoDam in the INPs using *D*-GAL4 (GMR12E09-GAL4 (Bayraktar and Doe, 2013; Figure S4A). NanoDam profiles binding only in a subset of the *D*-GAL4 expression pattern, in cells that also express the endogenously tagged TF (Figure 2A). We crossed *D-GAL4*; *UAS-NanoDam* to flies expressing endogenously tagged D-GFP, Grh-GFP, or Ey-GFP (Kudron et al., 2018). Genomic DNA was extracted from approximately 50 dissected brains per sample and processed as described previously for TaDa in order to generate libraries of fragments corresponding to TF bound regions (Marshall et al., 2016; Southall et al., 2013).

As a first step in searching for temporal TFs regulated by D, Grh, or Ey, we compared the NanoDam peaks for D, Grh, and Ey with one another throughout the genome and clustered them according to their aggregated binding intensities. Clustering revealed 6 different combinations of D, Grh, and Ey binding in INPs (Figures 2B and S2). Peaks in clusters showed the following: ND1 corresponded to strong D binding but minimal binding of Grh or Ey; ND2 peaks showed strong D and Grh binding; ND3, strong Grh binding; ND4, strong binding of D, Grh, and Ey; ND5, Ey binding; and ND6, minimal binding of all three TFs. This suggested complex regulatory relationships between these temporal factors and their target genes.

To determine the functional relevance of these ND clusters, we assigned the peaks within each cluster to the nearest transcriptional start sites of protein-coding genes. Within the lists we identified genes encoding TFs (Figures 2B and S2C) and hypothesized that some of these might be INP temporal TFs whose expression is regulated by D, Grh, or Ey.

### scRNA-seq of INPs and their progeny

**Figure 3:**
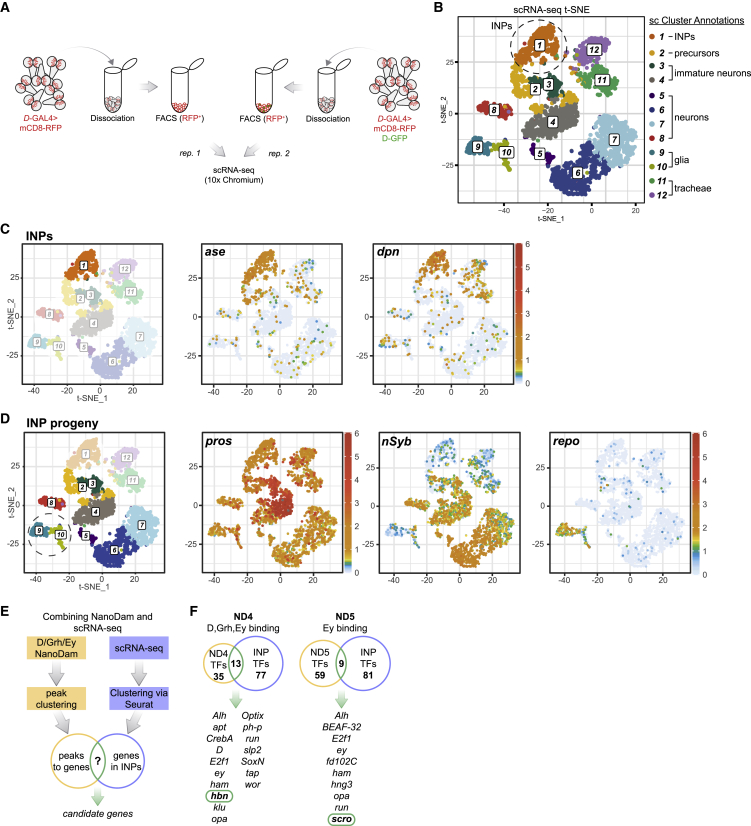
Combining NanoDam with scRNA-seq identifies *homeobrain* (*hbn*) and *scarecrow* (*scro*) as candidate temporal factors in INPs (A) Schematic overview of the experimental single-cell RNA sequencing (scRNA-seq) approach. We acquired 2 replicates: replicate 1 (rep.1) from brains carrying *D*-GAL4>mCD8-RFP (right) and replicate 2 (rep. 2) from brains carrying *D*-GAL4>mCD8-RFP and D-GFP (left). Brains were dissected at wandering third-instar stage, dissociated, and then cells were sorted based on RFP expression before being submitted for 10× chromium scRNA-seq. (B) t-distributed stochastic neighbor embedding (t-SNE) visualisation of 4,086 sorted single cells colored by cluster assignment and annotated based on previously known markers (see Figure S3). (C) t-SNE visualisation highlighting cluster 1 corresponding to INPs. Cluster 1 was designated as INPs due to enriched expression of *asense* (*ase*), *deadpan* (*dpn*), *earmuff* (*erm*) and *worniu* (*wor*) (see Figure S3). (D) Clusters 2–10 contain a mixture of INP progeny and express high levels of *Prospero* (*pros*). Clusters 2–8 express *neuronal synaptobrevin* (*nSyb*) and are thus classified as neurons. Clusters 9 and 10 (outline with dotted circle) correspond to glia due to high expression of *reversed polarity* (*repo*). (E) Workflow used to identify candidate temporal factors. (F) Candidate temporal transcription factors *homeobrain* (*hbn*) and *scarecrow* (*scro*) (outlined in green) were significantly differentially expressed in INPs (based on the scRNA-seq data) and identified in NanoDam cluster 4 (ND4) or NanoDam cluster 5 (ND5).

In order to identify genes whose expression was enriched in INPs, we carried out scRNA-seq of INPs and their progeny. We drove expression of membrane-targeted RFP (Pfeiffer et al., 2010) in INPs with *D*-GAL4 (Figure S4A). Dissected brains from wandering third-instar larvae were dissociated enzymatically and RFP-positive cells were isolated by FACS (Figure 3A). We recovered 4,086 single cells from approximately 230 brains across 2 biological replicates (Figures S3A and S3B) with 2,614 median genes detected per cell. We clustered the cells with Seurat (Butler et al., 2018; Stuart et al., 2019), generating 12 clusters that were visualized using a t-distributed stochastic neighbor embedding (t-SNE) plot (Figure 3B; Maaten and Hinton, 2008). Cluster identities were assigned based on known cell-type-specific markers: cluster 1 was designated as INPs (*dpn*, *wor, ase,* and *erm*) (Figures 3C and S3C); clusters 2–10 as INP progeny (GMCs, neurons, and glia); clusters 2–4 as immature neurons (*nSyb* and high *pros*); and clusters 9–10 corresponding as glial cells (*repo*) (Figure 3D). Cluster 2 also contains GMCs that do not express *nSyb* or *dpn* but have high levels of *ase* and *pros* (Figures 3C and 3D).

As expected, all three temporal factors *D*, *grh*, and *ey* were expressed in the INP cluster (Figure S3D). We also observed significant expression of *grh* in clusters 11 and 12 (Figure S3D), which, interestingly, did not show high levels of expression of brain-specific markers (Figures 3C and 3D) but instead were enriched for tracheal gene expression (Figure S3E). The expression of *grh* in these tracheal cell clusters is consistent with a previous study identifying a functional role for Grh in tracheal development (Yao et al., 2017). Thus, we excluded clusters 11 and 12 from our analysis to focus solely on INPs and their progeny.

### Identifying missing INP temporal factors

We determined which TFs in each NanoDam cluster were expressed in INPs by comparison with our scRNA-seq dataset (Figure 3E; Table S1). In addition to TFs known to be involved in INP cell identity (*ase*, *dpn*, *wor*) and those shown previously to regulate temporal identity (*D*, *grh*, *ey*, *opa*, *ham*), we found several candidate temporal TFs. We focused our attention on two factors that were expressed in a subset of INPs: the paired-like homeobox TF *homeobrain* (*hbn*) (Walldorf et al., 2000) and the NK-2 homeobox TF *scarecrow* (*scro*) (Zaffran et al., 2000; Figure 3F).

**Figure 4:**
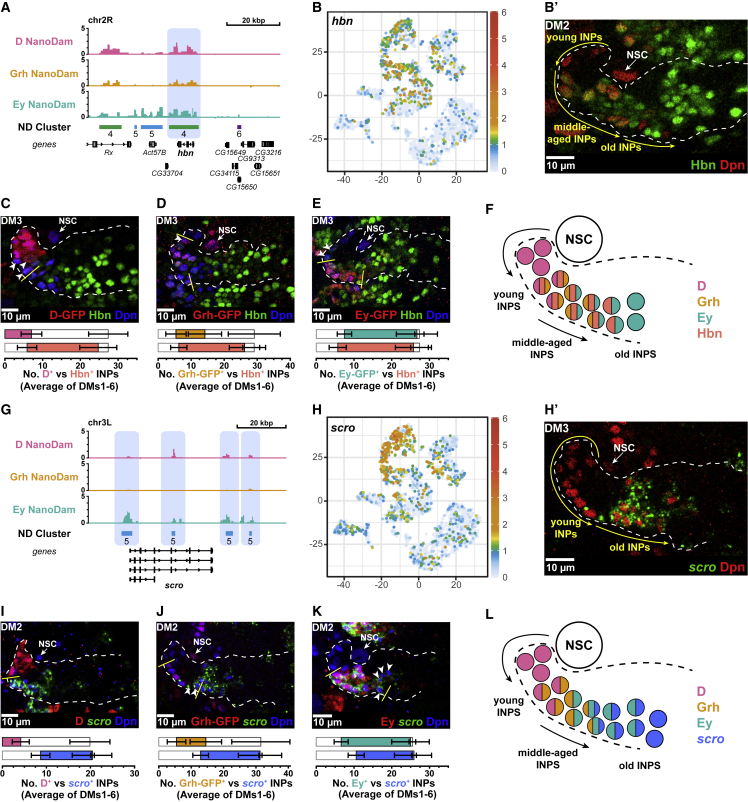
Temporal expression patterns of Homeobrain and *scarecrow* (A) NanoDam binding of D, Grh, and Ey across the *hbn* gene locus and nearby regions. The ND6 peak across *hbn* is highlighted. Binding intensities are shown as log_2_-fold enrichment of TF-GFP NanoDam over NanoDam only. (B) *hbn* is expressed in INPs. (B) t-SNE plot colored by *hbn* expression. (B′) Hbn (green) is not expressed in the type II NSC (white arrow) but is expressed in INPs (Dpn^+^ [red]) and progeny (Dpn^−^). Hbn expression begins across a broad domain of middle-aged INPs but is absent from the oldest INPs (Figure S4B for grayscale). (C) Overlap of D-GFP (red) and Hbn (green) in INPs (Dpn^+^ [blue]). Hbn expression begins at the end of the D temporal window. Only the oldest D^+^ INPs express Hbn (arrowheads) (Figure S4D for grayscale). Very few INPs expressed both D and Hbn and the few D^+^ Hbn^+^ INPs were found at the end of the D temporal window (Figure S4D′). (D) Hbn (green) overlaps with Grh^+^ (red) INPs. Arrowhead indicates a young Grh^+^ INP that does not express Hbn (Figure S4E for grayscale). (E) Hbn (green) expression begins before the Ey (red) window. Arrowheads indicate the youngest Hbn^+^ INPs that do not express Ey (Figure S4F for grayscale). (F) Proposed model for the expression pattern of Hbn and its relationship to D, Grh, and Ey expression, based on the quantifications in Figure S4. (G) NanoDam binding of D, Grh, and Ey across the *scro* gene locus. The 4 ND5 peaks are highlighted. Binding intensities are shown as log_2_-fold enrichment of TF-GFP NanoDam over NanoDam only. (H) *scro* is expressed in INPs. (H) t-SNE plots colored by *scro* expression. (H′) *scro* (green) is not expressed in the type II NSC (white arrow) or young INPs (Dpn^+^ [red]) but is expressed in old INPs (Dpn^+^) (Figure S4C for grayscale). (I) *scro* (green) does not overlap with D (red) in INPs (Dpn^+^ [blue]) (Figure S4G for grayscale). (J) The oldest Grh^+^ (red) INPs express *scro* (green) (arrowheads) (Figure S4H for grayscale). (K) *scro* (green) expression begins after the activation of Ey (red) in INPs. The oldest *scro*^+^ INPs do not express Ey (arrowheads) (Figure S4I for grayscale). (L) Proposed model for the expression pattern of *scro* and its relationship to D, Grh, and Ey expression, based on the quantifications in Figure S4. All quantifications represent an average across DM1–6. Error bars represent standard deviation. White dotted lines indicate *D*-GAL4>*mCD8-RFP* expression (which is expressed in INPs and their daughter cells) in single section confocal images. n = 6 brain lobes.

*homeobrain* clusters in ND4 (strong binding of *D*, *Grh*, and *Ey*; Figure 4A), suggesting that multiple members of the temporal cascade may regulate its expression. *hbn* was also highly enriched in INPs according to our scRNA-seq data (Figure 4B). Examining Hbn expression in type II lineages *in vivo* revealed that Hbn was expressed in middle-aged and old INPs but absent from NSCs and the youngest INPs (Figures 4B′–4F and S4B–S4F). In most type II lineages, Hbn was expressed in INPs concomitantly with Grh (Figures 4D and S4E) and prior to initiation of Ey expression (Figures 4E and S4F). Hbn expression was maintained throughout most of the Ey temporal window, except in the oldest INPs in the DM2 and DM3 lineages (Figure S4F). Therefore, Hbn expression defines a new temporal window extending from the end of D expression through the Ey expression window.

*scro* clusters in ND5 are bound strongly by Ey and weakly by D, but not by Grh, (Figure 4G) and is highly enriched in INPs (Figure 4H). We assayed *scro* expression in type II lineages *in vivo* by fluorescent *in situ* hybridization chain reaction (HCR) (Choi et al., 2010, 2014, 2016, 2018). *scro* was expressed in the oldest INPs in all DM lineages and absent from type II NSCs and young INPs (Figures 4H′ and S4G). *scro* was never co-expressed with D (Figures 4I and S4G), and only the oldest Grh^+^ INPs expressed *scro* (Figures 4J, S4H, and S4J). In all lineages (DM1–6) *scro* expression began after Ey and was maintained into the oldest INPs (Figures 4K and S4I). Therefore, *scro* expression defines the latest temporal window (Figure 4L).

### The middle-aged factor Hbn displays classic temporal factor interactions with Ey and *scro*

**Figure 5:**
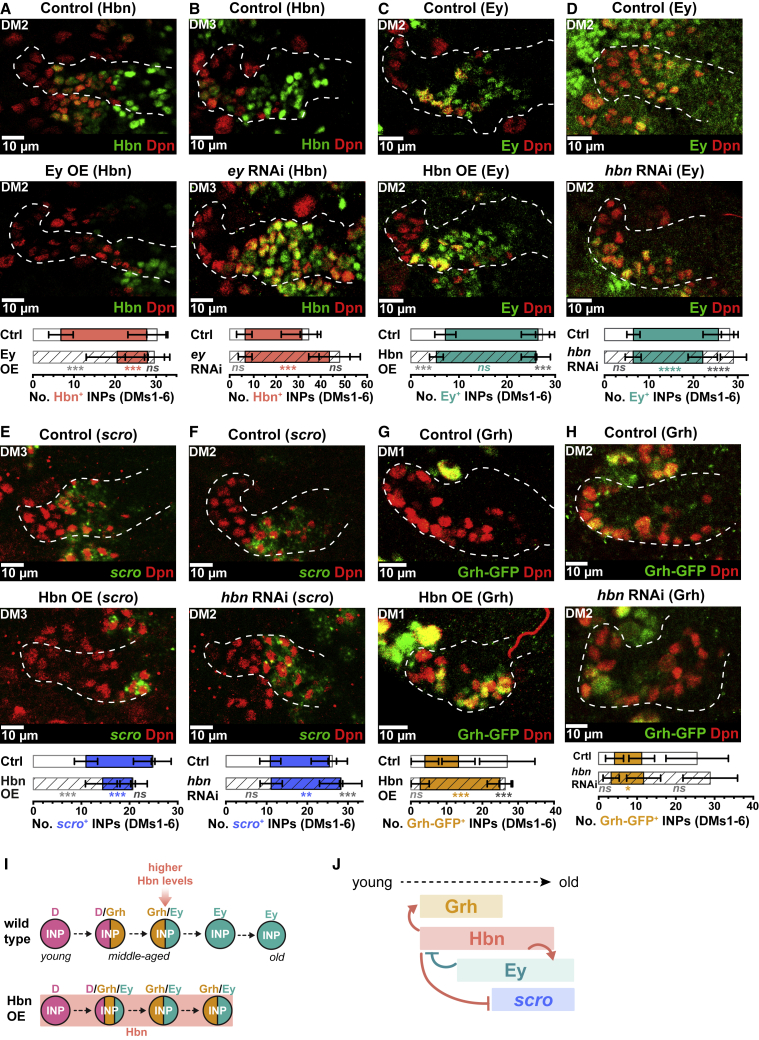
Homeobrain exhibits regulatory relationships typical of temporal factors (A) Ey misexpression represses Hbn (green) in INPs (Dpn^+^ [red]). Mann-Whitney test p < 0.001, ^∗∗∗^; p < 0.001, ^∗∗∗^; p = 0.08, ns (Figure S5A for grayscale). (B) *ey* RNAi results in more Hbn^+^ (green) in INPs (Dpn^+^ [red]). Control is *w*^*1118*^. Mann-Whitney test p = 0.93, ns; p < 0.001, ^∗∗∗^; p = 0.08, ns (Figure S5C for grayscale). (C) Hbn misexpression precociously activates Ey (green) in INPs (Dpn^+^). Mann-Whitney test p < 0.001, ^∗∗∗^; p = 0.15, ns; p < 0.001, ^∗∗∗^ (Figure S5E for grayscale). (D) Hbn RNAi leads to less Ey (green). Mann-Whitney test p = 0.9838, ns; p < 0.0001, ^∗∗∗∗^; p = 0.0001, ^∗∗∗∗^ (Figure S5G for grayscale). (E) Hbn misexpression represses *scro* (green). Mann-Whitney test p < 0.001, ^∗∗∗^; p < 0.001, ^∗∗∗^; p = 0.73, ns (Figure S5H for grayscale). (F) Hbn knockdown leads to an increase in *scro* (green). Mann-Whitney test p = 0.83, ns; p = 0.002, ^∗∗^; p = 0.0005, ^∗∗∗^ (Figure S5I for grayscale). (G) Hbn misexpression leads to ectopic activation of Grh (green) in INPs (Dpn^+^ [red]). Control image is a projection over 8 μm and Hbn OE is a projection over 29 μm. Mann-Whitney test p = 0.13, ns; p < 0.001, ^∗∗∗^; p < 0.001, ^∗∗∗^ (Figure S6B for grayscale). (H) Loss of hbn results in a slight decrease of Grh expression. Mann-Whitney test (averaged across DM1–6) p = 0.0642, ns; p = 0.0239, ^∗^; p = 0.0822, ns (Figure S6D for grayscale). (I) Summary of the phenotype of Hbn misexpression. Note in wild type the strength of Hbn expression is higher later in the cascade. (J) Summary of the regulatory relationships of Hbn in the temporal cascade. All quantifications represent an average across DM1–6. Error bars represent standard deviation. Single section confocal images unless stated otherwise. White dotted lines indicate *D*-GAL4>*mCD8-RFP* expression. n = 6 brain lobes.

Next, we investigated the regulatory interactions between the known INP temporal factors and Hbn and Scro. Hbn is expressed in middle-aged INPs (Figure 4F). Ey binds the *hbn* locus and is expressed in INPs after Hbn. Given that temporal factors are thought to repress transcription of the factor that precedes them, we tested whether Ey repressed Hbn expression. We found that ectopic expression of Ey in all INPs resulted in a significant reduction in Hbn expression (Figures 5A, S5A, and S5A′), whereas knocking down *ey* expression in INPs (Figure S5B) extended the Hbn window into the oldest INPs without affecting early Hbn expression (Figures 5B, S5C, and S5C′). This was true of all lineages except DM5, in which the oldest INPs remained Hbn^−^ (Figures S5C′ and S5D). Knocking down *ey* with RNAi also increased the number of INPs in all DM lineages, as has been reported previously (Bayraktar and Doe, 2013). Therefore, Ey terminates Hbn expression in old INPs (Figure 5I), a regulatory interaction characteristic of temporal factors.

Another predicted regulatory relationship between temporal factors is that each activates expression of the subsequent factor and represses the next plus one. This would suggest that Hbn should activate Ey expression and repress Scro. We found that misexpression of Hbn in INPs activated the Ey temporal window precociously (Figures 5C, S5E, and S5E′), whereas a loss of *hbn* led to a reduction of the Ey window without affecting onset (Figures 5D, S5G, and S5G′). Aside from its effects on Ey, misexpression of Hbn also resulted in a reduction in *scro* (Figures 5E, S5H, and S5H′). Consistent with these results, knockdown of *hbn* by RNAi (Figure S5F) led to an increase in *scro* expression (Figures 5F, S5I, and S5I′). We conclude that Hbn activates Ey and represses *scro* (Figures 5H and 5I), the type of behavior attributed to classically defined temporal TFs, exemplified in the embryonic temporal cascade (Isshiki et al., 2001).

### Hbn is sufficient to activate the middle-aged temporal factor Grh

We observed that the onset of Hbn expression coincided with the start of Grh expression. Furthermore, ectopic expression of Hbn resulted in concomitant expression of Grh, and we noticed that INPs with stronger Hbn staining signal also correlated with higher Grh signal (Figure S6A). We postulated that these two temporal factors shared a regulatory relationship. We found that ectopic Hbn expression was sufficient to activate Grh precociously and to extend its expression window in all DM lineages (Figure 5G), even in DM1, which normally does not express Grh. Grh was induced in almost all INPs in the DM1 lineage (Figure S6B′). Hbn is normally expressed at lower levels in DM1 than in others, suggesting that Hbn may activate Grh in a dosage-dependent manner. Forced expression of Hbn in DM lineages drove INPs toward a “middle-aged fate”: most INPs remained D^−^ Grh^+^ Ey^+^ (Figure 5I). We also observed that lineages DM1–4 and DM6 had significantly fewer INPs (Figure S6C), suggesting that Hbn may regulate cellular longevity during the middle-aged window. Knockdown of *hbn* expands the Grh window in DM2 and 3, though the effect is not as striking (Figures 5H, S6D, and S6D′) and may be due to a reduction of Ey, which normally restricts Grh expression. Taken together, our results demonstrate that Hbn is a temporal TF that activates Grh and Ey expression and represses *scro* to aid the progress of temporal transitions (Figure 5J).

We also investigated whether Grh had reciprocal regulatory interactions with Hbn. Neither misexpression nor loss of Grh significantly impacted the Hbn window (Figures S6E–S6F′). Loss of Grh in DM3 resulted in an earlier termination of Hbn expression (Figure S6F′). These results suggest that Grh is not required for the onset of Hbn expression and may have a minor role in the maintenance of the Hbn window.

### *scro* acts in a negative feedback loop with Ey

**Figure 6:**
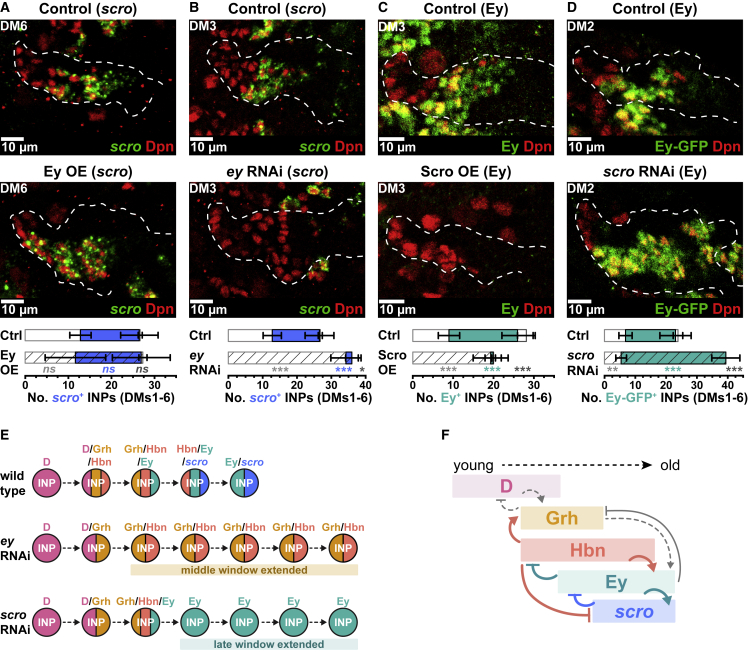
Scarecrow is a late temporal factor that represses Eyeless (A) Ey OE precociously activates *scro* (green) in INPs (Dpn^+^ [red]). Mann-Whitney test p = 0.12, ns; p = 0.44, ns; p = 0.60 (Figure S6G for grayscale). (B) *ey* RNAi leads to loss of *scro* (green) in INPs (Dpn^+^ [red]). Mann-Whitney test p < 0.001, ^∗∗∗^; p < 0.001, ^∗∗∗^; p = 0.02, × (Figure S6H for grayscale). (C) Misexpression of Scro leads to loss of Ey (green) in INPs (Dpn^+^ [red]). Mann-Whitney test p < 0.001, ^∗∗∗^ for all (Figure S6I for grayscale). (D) *scro* RNAi leads to an increase in the number of Ey^+^ (green) INPs (Dpn^+^ [red]). Mann-Whitney test p = 0.007, ^∗∗^; p < 0.001, ^∗∗∗^; p < 0.001, ^∗∗∗^ (Figure S6J for grayscale). (E) Summary of *scro* loss of function phenotype compared to wild type and *ey* loss of function. (F) Summary of the regulatory relationships between Hbn, *scro*, Ey, and Grh. The gray arrows indicate previously established regulatory relationships. All quantifications represent an average across DM1–6. Error bars represent standard deviation. Single section confocal images unless stated otherwise. White dotted lines indicate *D*-GAL4>*mCD8-RFP* expression. n = 6 brain lobes.

We found that *scro* expression was restricted to the oldest INPs, overlapping and extending beyond the Ey expression window (Figure 4L). NanoDam revealed that Ey was bound at the *scro* locus, leading us to hypothesize that Ey might activate *scro* transcription in INPs. Ectopic expression of Ey was sufficient to activate *scro* precociously in all DM lineages except DM2 and 3, where a reduction in *scro* was observed (Figures 6A, S6G, and S6G′). Conversely, knocking down *ey* expression in INPs lead to an almost complete loss of *scro* expression (Figures 6B, S6H, and S6H′). We conclude that Ey is both necessary and sufficient to activate *scro* expression and that Ey is likely to act directly.

Given the reciprocal regulatory interactions observed between temporal factors, we tested whether *scro*, as the last identified temporal factor in INPs, represses Ey expression to terminate the Ey temporal window. In support of this hypothesis, we found that ectopic expression of Scro resulted in the loss of Ey (Figures 6C, S6I, and S6I′). Next, we assessed whether the loss of *scro* in INPs would affect Ey expression. Using two independent *scro* RNAi constructs that knocked down expression effectively (Figure S6K), we found that the loss of *scro* extended the Ey temporal window (Figures 6D, S6J, and S6J′), without affecting the number of D^+^ or Grh^+^ INPs (Figures S6L, S6L′, S6M, and S6M′). Therefore, Scro is necessary and sufficient to close the Ey temporal expression window.

Interestingly, we observed that lineages lacking *scro* contained significantly more INPs than controls, suggesting an effect on longevity (Figure S6N). It had been reported previously that the loss of Ey lead to an increased number of INPs (Bayraktar and Doe, 2013), comparable to the numbers we observed after the loss of *scro* (Figure S6N). The effects on temporal patterning, however, were distinct. Removing Ey extended the middle-aged temporal window (INPs remained Grh^+^/Hbn^+^) (Figure 6E; Bayraktar and Doe, 2013). By contrast, in the absence of *scro*, INPs progressed through the middle-aged window, and instead, the late temporal window was extended (INPs remained Ey^+^) (Figures 6D and 6E). Together, our results indicate that *scro* is activated directly by Ey in INPs and that Scro restrains the Ey temporal window at the end of the temporal cascade (Figure 6F).

### Hbn and Scro act as temporal factors in the developing visual system

**Figure 7:**
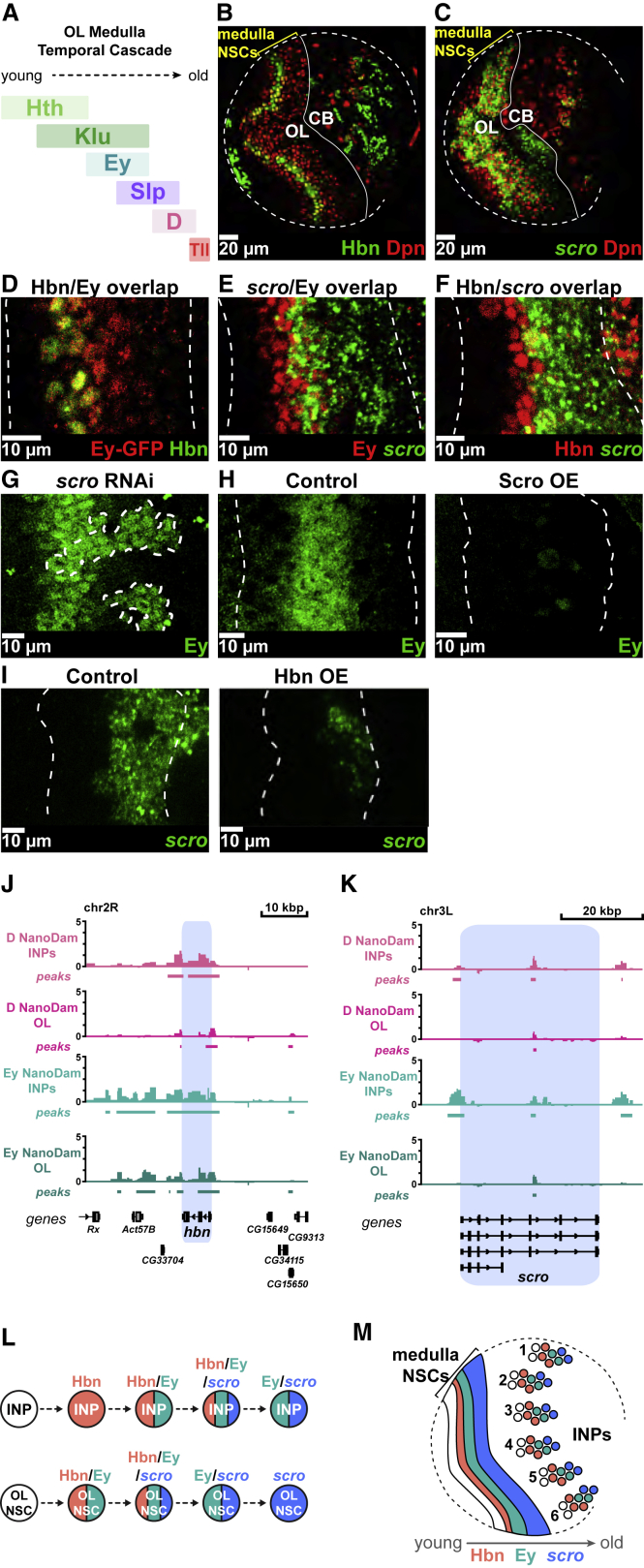
Hbn and *scro* are expressed in temporal windows in the optic lobe temporal cascade (A) Schematic showing the temporal cascade of the optic lobe (OL) medulla NSCs. (B) Hbn (green) is expressed in the OL medulla NSCs (Dpn^+^ [red] and highlighted with yellow bracket) in addition to the central brain (CB) (Figure S7A for grayscale). (C) *scro* (green) is expressed in the OL medulla NSCs (Dpn^+^ [red] and highlighted with yellow bracket) in addition to the CB (Figure S7B for grayscale). (D) Hbn (green) expression coincides with the start of the Ey temporal window (Ey-GFP^+^, [red]) in medulla NSCs (Figure S7F for grayscale). (E) *scro* (green) expression begins in the second half of the Ey window (Ey, red) (Figure S7G for grayscale). (F) *scro* (green) is expressed in the oldest Hbn^+^ (red) OL medulla NSCs (Figure S7H for grayscale). (G) Loss of *scro* extends Ey expression (green) in medulla NSCs. Clones expressing *scro* RNAi^TRiP^ are indicated with white dotted outlines. (H) Ectopic Scro expression in OL NSCs (*insc*-GAL4>UAS-s*cro*) results in the loss of the Ey temporal window (green). (I) Ectopic Hbn expression in OL NSCs (*insc*-GAL4>UAS-*hbn*) leads to a reduction of s*cro* expression (green). (J) NanoDam binding profiles of D and Ey across the *hbn* gene locus (blue) in INPs and OL NSCs. *ogre*-GAL4 was used to drive NanoDam in OL NSCS in combination with D-GFP or Ey-GFP. Binding intensities are shown as log_2_-fold enrichment of TF-GFP NanoDam over NanoDam only. (K) NanoDam binding profiles of D and Ey across the *scro* gene locus (blue) in INPs and OL NSCs. Binding intensities are shown as log_2_-fold enrichment of TF-GFP NanoDam over NanoDam only. (L) The order of Hbn, Ey, and *scro* expression is conserved in the INP and OL medulla NSC temporal cascades. Single section confocal images. Dotted white lines in (B) and (C) outline the edges of the brain lobes; (D)–(I) indicate the region containing medulla NSCs. n = 6 brain lobes. (M) The conserved expression of Hbn, Ey, and *scro* in medulla NSCs and INPs of the DM type II lineages.

Temporal patterning also regulates neuronal diversity in the developing OL, where D and Ey are expressed temporally in NSCs in the medulla of the OL (Figure 7A; Bayraktar and Doe, 2013; Li et al., 2013; Suzuki et al., 2013). Intriguingly, we found Hbn and *scro* were also expressed in subpopulations of medulla NSCs (Figures 7B, 7C, and S7A–S7E). As we had observed in INPs, we found that Hbn, Ey, and *scro* were expressed sequentially in medulla NSCs (Figures 7D–7F and S7F–7H). Interestingly, we could show that several of the regulatory interactions we discovered in INPs were conserved in medulla NSCs. We found that *scro* was necessary and sufficient for repression of Ey in NSCs (Figures 7G–7H), indicating that termination of the Ey temporal window by *scro* is conserved between INPs and the OL. In addition, misexpression of Hbn dramatically reduced *scro* expression, fulfilling the “repression of next plus one” rule (Figure 7I). A comparison of Ey and D binding in INPs and the OL revealed slightly different patterns at the *hbn* and *scro* loci (Figures 7J–7K): D binds strongly at the *hbn* locus in INPs, but more weakly in the OL. This may reflect differential regulation in the two cell types. For example, in contrast to INPs, in the OL D is expressed after Hbn, and the D and Hbn temporal windows do not overlap (Figure S7E). We conclude that the temporal expression cascade, from Hbn to Ey to *scro*, is conserved in progenitors that generate both the central complex of the brain and the visual processing system (Figures 7L and 7M). This is particularly striking as INPs and OL NSCs are distinct progenitors with different origins, the former born from asymmetrically dividing type II NSCs and the latter derived from symmetrically dividing neuroepithelial cells (Egger et al., 2007).

## Discussion

Temporal patterning leads to the generation of neuronal diversity from a relatively small pool of neural stem or progenitor cells. Temporal regulation is achieved by the restricted expression of temporal TFs within precise developmental windows. The onset and duration of each temporal window in these cells must be regulated tightly in order for the appropriate subtypes of neurons to be generated at the correct time to establish functional neuronal circuits.

Here, we focused on the INPs of the type II NSC lineages that generate the central complex of the *Drosophila* brain. The neural diversity generated by the INP lineages, suggested that unknown temporal factors remained to be discovered. With the expectation that these factors would be the transcriptional targets of the known temporal factors, we developed NanoDam to profile the binding targets of D, Grh, and Ey. NanoDam enables both spatial and temporal specificity, at the cellular level and within defined temporal windows.

NanoDam permits genome-wide profiling of any endogenously tagged chromatin-binding protein after a simple genetic cross, bypassing the need to generate transgenes expressing Dam-fusion proteins, or the requirement for specific antisera, cross-linking, or cell isolation. Binding profiles within a subset of the protein’s expression pattern can be achieved by expressing NanoDam with specific GAL4 drivers (Figure 1D). To date, collaborative efforts have produced more than 3,900 *Drosophila* lines expressing GFP-tagged proteins in their endogenous patterns (Table S2). Approximately 93% of all TFs have been GFP-tagged in lines that are publicly available at stock centres. Lines that are not yet available can be rapidly generated by CRISPR-Cas9-mediated tagging. NanoDam can be readily adapted for use in other organisms to facilitate easier *in vivo* profiling experiments, as we have demonstrated previously for TaDa (Cheetham et al., 2018; Aloia et al., 2019). The growing library of nanobodies that target proteins with specific post-translational modifications, such as histone modifications, or tags other than GFP enables NanoDam to be readily applied to profile a broad array of chromatin-binding proteins.

By combining the power of NanoDam with scRNA-seq, we were able to identify *scro* and *hbn* as temporal factors in the INPs. The mammalian homolog of *ey* (*Pax6*), *hbn* (*Arx*), and *scro* (*Nkx2.1*) are restricted to distinct progenitor populations in the developing mouse forebrain (Colombo et al., 2004; Götz et al., 1998; Sussel et al., 1999). We found that *scro* regulates the late INP identity by repression of Ey. Interestingly, the loss of *Nkx2.1* in the mouse forebrain leads to aberrant expression in ventral regions of the dorsal factor *Pax6* (Sussel et al., 1999), suggesting that the repressive relationship between *scro* and *ey* may be conserved between *Nkx2.1* and *Pax6*.

Not all relationships appear to be conserved, however. We found that Hbn promotes progression through the middle-aged temporal stage and that maintenance of the middle-aged temporal window is regulated in part by interactions between Hbn and Grh. *Arx* mutant mice exhibit loss of upper layer (later-born) neurons but no change in the number of lower layer (early-born) neurons (Colasante et al., 2015).

Intriguingly, both *scro* and *hbn* were also temporally expressed in OL NSCs (Figures 7B and 7C), and the regulatory relationships between *scro* and Ey appeared to be conserved. This suggests that similar regulatory strategies may be shared between NSCs or progenitor cells in order to regulate longevity and neuronal subtype production. The remarkable conservation of the regulatory interactions of *scro* in two different progenitor cell types with different origins in the *Drosophila* brain may also be translated to the context of mammalian neurogenesis, highlighting the possibility of a more generalized regulatory network used by stem and progenitor cells to regulate cell fate, progeny fate, and proliferation.

The type II lineages in *Drosophila* divide in a similar manner to the oRGs that have been attributed to the rapid evolutionary expansion of the neocortex seen in humans and other mammals (Fietz et al., 2010; García-Moreno et al., 2012; Hansen et al., 2010; Wang et al., 2011). Interestingly, oRGs show a shortened cell-cycle length in primates (Penisson et al., 2019) in comparison to rodent progenitors, which increase cell-cycle duration as development progresses (Betizeau et al., 2013). We found that manipulation of *scro* and *hbn* levels affects INP numbers. The loss of *scro* results in more INPs within a lineage, similar to the phenotype observed upon loss of Ey (Bayraktar and Doe, 2013). Ey activates *scro* in the temporal cascade, and it is likely that the increase in INPs upon loss of Ey is due to the loss of *scro* expression. Clonal analysis suggests that the increase in INP numbers is not due to aberrant symmetrical division (Bayraktar and Doe, 2013), suggesting that temporal factors have an effect on INP longevity. It would be interesting to investigate whether oRGs use temporally expressed factors to control longevity and cell-cycle dynamics at different developmental stages in order to regulate neuronal subtype generation during neocortex development.

There is significant heterogeneity between the type II lineages, and our study has identified differences in the regulatory relationships of *hbn* and *scro*. For example, misexpression of Ey leads to an increase in *scro* in all lineages except DM2 and 3, where *scro* expression is reduced. The heterogeneity between lineages may be a consequence of variations in combinatorial binding of temporal factors, as our NanoDam data indicate. The diversity of INPs and differences between lineages may be further investigated through lineage-specific or higher resolution scRNA-seq (Michki et al., 2021). Although INPs share temporal factors, different DM lineages display subtle to striking differences when the temporal cascade is manipulated, demonstrating the likelihood that each DM employs unique temporal cascades. Combinatorial binding would enable more complex regulatory interactions that could refine or subdivide temporal windows in the INPs.

### Limitations

In this study, we have utilised GFP-tagged TFs for NanoDam profiling. These GFP-tagged proteins retain their ability to bind DNA, as revealed by their specific genome-wide binding profiles. Moreover, Grh-GFP and Ey-GFP recapitulate the endogenous expression patterns in INPs and have been shown previously to have no effect on INP temporal windows (Abdusselamoglu et al., 2019). In rare cases where GFP might interfere with the DNA-binding ability of the protein of interest, alternative nanotags (Xu et al., 2022) can be used, as NanoDam is not limited to GFP tags.

In this study, small populations of cells were profiled with NanoDam (roughly 10% of cells labeled by D-GAL4>UAS-mCD8-RFP are INPs, of which only a subset express D/Grh/Ey). The minimum number of cells needed for NanoDam remains to be determined, though factors intrinsic to the protein, such as binding affinity or nuclear concentration, may influence this.

## STAR★Methods

### Key resources table

**Table:** 

REAGENT OR RESOURCE	SOURCE	IDENTIFIER
**Antibodies**		

Guinea pig anti-D	Gift from Alex Gould, Crick Institute	N/A
Guinea pig anti-Dpn	Caygill and Brand (2017)	N/A
Rat anti-Dpn	Abcam	11D1BC7; RRID: AB_2687586
Rabbit anti-Ey	Gift from Uwe Walldorf, Universität des Saarlandes	N/A
Chicken anti-GFP	Abcam	RRID: AB_300798
Rat anti-Grh	Baumgardt et al. (2009)	N/A
Rabbit anti-Hbn	Gift from Uwe Walldorf, Universität des Saarlandes	N/A
Goat anti-chicken Alexa Fluor 488	Invitrogen	REF A11039; RRID: AB_2534096
Goat anti-guinea pig Alexa Fluor 568	Invitrogen	REF A11075; RRID: AB_2534119
Goat anti-guinea pig Alexa Fluor 633	Invitrogen	REF A21105; RRID: AB_2535757
Goat anti-rabbit Alexa Fluor 488	Invitrogen	REF A11034; RRID: AB_2868494
Goat anti-rabbit Alexa Fluor 405	Invitrogen	REF A31556; RRID: AB_221605
Goat anti-rabbit Alexa Fluor 633	Invitrogen	REF A21071; RRID: AB_2535732
Goat anti-rat Alexa Fluor 568	Invitrogen	RRID: AB_2534121
Goat anti-rat Alexa Fluor 633	Invitrogen	REF A21094; RRID: AB_2535749
Donkey anti-guinea pig Dylight 405-conjugated AffiniPure	Jackson Immunoresearch	706-475; RRID: AB_2340470

**Chemicals, peptides, and recombinant proteins**		

16% Formaldehyde Solution (w/v)	ThermoFisher Scientific	28908
Papain from papaya latex	Sigma Life Sciences	P3375, LOT SLBR9817V
Collagenase type I	Merck Millipore	SCR103
Schneider’s Insect Medium	Sigma Aldrich	S9895
Triton X-100	Sigma Aldrich	T9284, Lot SLBF2001V
Tween 20	Sigma Aldrich	P7949 Lot 109K01021
Probe Hybridisation Buffer	Molecular Instruments	N/A
Probe Wash Buffer	Molecular Instruments	N/A
Amplification Buffer	Molecular Instruments	N/A
Vectashield Antifade Mounting Medium	Vector Laboratories	H-1000-10
SlowFade Gold Antifade Reagent	Invitrogen	Cat#S36936
SYTOX Blue Dead Cell Stain	Invitrogen	Cat#S34857

**Critical commercial assays**		

10X Chromium (v2 chemistry kit) Gene Expression Analysis	10X Genomics	N/A

**Deposited data**		

scRNA-seq data (raw and processed files)	This study	GEO: GSE190210
NanoDam data (raw and processed files)	This study	GEO: GSE190210

**Experimental models: Organisms/strains**		

*D*-GAL4	BDSC	48510 (GMR12E09)
*insc*-GAL4	Luo et al. (1994)	MZ1407
*ogre*-GAL4	BDSC	49340 (GMR29C07)
*wor*-GAL4	Albertson et al. (2004)	N/A
*tub*-GAL80^ts^	BDSC	7019
AyGAL4, UAS-GFP	BDSC	RRID: BDSC_4411
10xUAS-*IVS-mCD8-RFP*	BDSC	RRID: BDSC_32219
UAS-*mCD8-GFP*	BDSC	RRID: BDSC_5130
UAS-*Dicer2*	VDRC	60008
*hsFLP*^122^	N/A	N/A
*cph*::YFP	CPTI	RRID: DGGR_115236
D-GFP	BDSC	RRID: BDSC_66758
Grh-GFP	BDSC	RRID: BDSC_42272
Ey-GFP	BDSC	RRID: BDSC_42271
UAS-D	BDSC	RRID: BDSC_8861
UAS-*ey*	BDSC	RRID: BDSC_6294
UAS-*grh*	BDSC	RRID: BDSC_42227
UAS-*hbn*	This study	N/A
UAS-*scro*	This study	N/A
UAS-*ey*-RNAi	VDRC	106628
UAS-*grh*-RNAi	VDRC	101428
UAS-*hbn*-RNAi^CDS2^	This study	N/A
UAS-*hbn*-RNAi^3UTR^	This study	N/A
UAS-*mCherry*-RNAi	BDSC	RRID: BDSC_35785
UAS-*scro*-RNAi	BDSC	RRID: BDSC_33890
UAS-*scro*-RNAi^CDS^	This study	N/A
UAS-*NanoDam*	This study	N/A
*w*^*1118*^		N/A

**Oligonucleotides**		

**vhhGFP4 Forward Primer** AGTGGCGGTGGGCCAAAAA AGAAAAGAAAAAGTCCGCGGC AAATGGATCAAGTCCAACTGGTGG	This study	N/A
**vhhGFP4 Reverse Primer** ATGTCACACCACAGAAGTAAGGTTCC TTCACAAAGATCCTTAGCTGGAGACG GTGACCTG	This study	N/A
**Hbn Coding Sequence Forward Primer** AGATGAATTCATGATGACCACGACGACCTCG	This study	N/A
**Hbn Coding Sequence Reverse Primer** ATGACTCGAGTCAGTCCTCGCCCTTGGTG	This study	N/A
***scro-RA* coding sequence Forward Primer** TACCAGGAATTCATGTCATCGCACGGCCTTGCTTAC	This study	N/A
***scro-RA* coding sequence Reverse Primer** TAGTATGCGGCCGCTTACCATGCCCGGC CTTGTAAGGG	This study	N/A
**Short hairpins for *hbn*-shRNA**^**CDS2**^**(Forward)** ctagcagtGTTCGAAGATCTCGCACAACAtagttata ttcaagcataTGTTGTGCGAGATCTTCGAACgcg	This study	N/A
**Short hairpins for *hbn*-shRNA**^**CDS2**^**(Reverse)** aattcgcGTTCGAAGATCTCGCACA ACAtatgcttgaatataactaTGTTGTGCGAGATC TTCGAACactg	This study	N/A
**Short hairpins for *hbn*-shRNA**^**3UTR**^**(Forward)** ctagcagtGATTCGTTATGTACATATATAtagttatat tcaagcataTATATATGTACATAACGAATCgcg	This study	N/A
**Short hairpins for *hbn*-shRNA**^**3UTR**^**(Reverse)** aattcgcGATTCGTTATGTACATATATAtatgcttgaa tataactaTATATATGTACATAACGAATCactg	This study	N/A
**Short hairpins for *scro*-shRNA**^**5UTR**^**(Forward)** ctagcagtAGTGGATATTCATAATAAATTtagttata ttcaagcataAATTTATTATGAATATCCACTgcg	This study	N/A
**Short hairpins for *scro*-shRNA**^**5UTR**^**(Reverse)** aattcgcAGTGGATATTCATAATAAATTtatgcttgaa tataactaAATTTATTATGAATATCCACTactg	This study	N/A
**Short hairpins for *scro*-shRNA**^**CDS**^**(Forward)** ctagcagtGCCCAGGTGTACAGACCTATTtagtta tattcaagcataAATAGGTCTGTACACCTGGGCgcg	This study	N/A
**Short hairpins for *scro*-shRNA**^**CDS**^**(Reverse)** aattcgcGCCCAGGTGTACAGACCTATTtatgcttg aatataactaAATAGGTCTGTACACCTGGGCactg	This study	N/A
*scro* HCR probe set (B3)	Molecular Instruments	N/A
B3 Amplifier 647	Molecular Instruments	N/A
B3 Amplifier 488	Molecular Instruments	N/A

**Recombinant DNA**		

pUAST-mCherry-Dam-vhhGFP4 (pUAST-NanoDam)	This study	N/A
pUAST-mCherry-Dam	Southall et al. (2013)	N/A
pWALIUM20 Vector	Perkins et al. (2015)	N/A
UAST-attB	Bischof et al. (2007)	N/A

**Software and algorithms**		

Fiji	Schindelin et al. (2012)	https://imagej.net/software/fiji/
FastQC (v0.11.5)	Andrews (2010)	https://www.bioinformatics.babraham.ac.uk/projects/fastqc/
damidseq_pipeline	Marshall and Brand (2015)	https://github.com/AHBrand-Lab/DamID_scripts
slurm workload manager (v15.08.13)	SchedMD	https://slurm.schedmd.com/download.html
bowtie2 (v2.3.4.1)	Langmead and Salzberg (2012)	https://sourceforge.net/projects/bowtie-bio/files/bowtie2/2.3.4/
bedGraphToBigWig (v4)	UCSC	https://www.encodeproject.org/software/bedgraphtobigwig/
MACS2 (v2.1.2)	Zhang et al. (2008)	https://pypi.org/project/MACS2/
bedtools (v2.26.0)	Quinlan and Hall (2010)	https://github.com/arq5x/bedtools2
stats (v3.6.1)	R	https://stat.ethz.ch/R-manual/R-devel/library/stats/html/00Index.html
factoextra (v1.0.5)	R	https://cran.r-project.org/web/packages/factoextra/index.html
clValid (v0.6-6)	R	https://cran.r-project.org/web/packages/clValid/index.html
mclust (v5.4.5)	R	https://cran.r-project.org/web/packages/mclust/index.html
cluster (v2.0.7-1)	R	https://cran.r-project.org/web/packages/cluster/index.html
GraphPad Prism 8	GraphPad Software	https://www.graphpad.com/
Seurat (v2.3.4)	Butler et al. (2018); Stuart et al. (2019)	https://cran.r-project.org/src/contrib/Archive/Seurat/
Adobe Illustrator (v25.4.2)	Adobe	https://www.adobe.com/products/illustrator.html
Cell Ranger (v2.2.1)	10X Genomics	https://support.10xgenomics.com/single-cell-gene-expression/software/downloads/latest
Integrative Genomic Viewer (IGV)	Robinson et al. (2011)	https://software.broadinstitute.org/software/igv/download
R	R-project	https://cran.ma.imperial.ac.uk/
RStudio	RStudio	https://www.rstudio.com/products/rstudio/download/
damMer.py	This study	https://github.com/AHBrand-Lab/NanoDam_analysis
damMer_tracks.py	This study	https://github.com/AHBrand-Lab/NanoDam_analysis
damMer_peaks.py	This study	https://github.com/AHBrand-Lab/NanoDam_analysis
**genomewide_correlation.Rmd**	This study	https://github.com/AHBrand-Lab/NanoDam_analysis
**signal_enrichment.Rmd**	This study	https://github.com/AHBrand-Lab/NanoDam_analysis
**create_annotations.Rmd**	This study	https://github.com/AHBrand-Lab/NanoDam_analysis
**cluster_peaks.Rmd**	This study	https://github.com/AHBrand-Lab/NanoDam_analysis
**annotate_peaks.Rmd**	This study	https://github.com/AHBrand-Lab/NanoDam_analysis
**scRNAseq_analysis.Rmd**	This study	https://github.com/AHBrand-Lab/NanoDam_analysis

**Other**		

Cell Sorter	Sony	SH800Z
Illumina HiSeq 4000	Illumina	N/A
Illumina HiSeq 1500	Illumina	N/A

### Resource availability

#### Lead contact

Further information and requests for resources and reagents should be directed to and will be fulfilled by the lead contact, Andrea Brand (ahb1000@cam.ac.uk).

#### Materials availability

Plasmids and fly stocks generated in this study are available upon request.

### Experimental model details

#### Fly Stocks

*Drosophila melanogaster* were reared in cages at 25 °C. Embryos were collected on yeasted apple juice plates. For experiments involving GAL80^ts^ embryos were kept at 18 °C until hatching. After hatching, larvae were transferred to a yeasted food plate and reared to wandering third larval instar stage before dissection.

The following lines were used to drive transgenes under the control of UAS in a spatially and temporally restricted manner: *D*-GAL4 (GMR12E09-GAL4, BDSC 48510), *insc*-GAL4 (*GAL4*^*MZ1407*^) (Luo et al., 1994), *ogre*-GAL4 (GMR29C07-GAL4, BDSC 49340), *wor*-GAL4 (Albertson et al., 2004), *tub*-GAL80^ts^ (BDSC 7019), Ay-GAL4, UAS-GFP (BDSC 4411), 10xUAS-*IVS-mCD8-RFP* (BDSC 32219), UAS-mCD8-GFP (BDSC 5130), UAS-*Dicer2* (VDRC 60008) and *hsFLP*^122^. The fusion proteins used were: *cph*::YFP (CPTI-001740, PMF and AHB, in preparation), D-GFP (BDSC 66758), Grh-GFP (BDSC 42272), Ey-GFP (BDSC 42271). The misexpression lines used were: UAS-D (BDSC 8861), UAS-*ey* (BDSC 6294), UAS-*grh* (BDSC 42227), UAS-*hbn* (this study), UAS-*scro* (this study). The RNAi lines used were: UAS-*ey*-RNAi (VDRC 106628), UAS-*grh*-RNAi (VDRC 101428), UAS-*hbn*-RNAi^CDS2^ (this study), UAS-*hbn*-RNAi^3UTR^ (this study), UAS-*mCherry*-RNAi (BDSC 35785), UAS-*scro*-RNAi (BDSC 33890) and UAS-*scro*-RNAi^CDS^ (this study). The generation of UAS-*NanoDam* is described below. *w*^*1118*^ was used for NanoDam control experiments and as a reference stock for functional experiments.

### Method details

#### Generation of expression constructs

pUAST-mCherry-Dam-vhhGFP4 (pUAST-NanoDam) was generated by PCR amplifying vhhGFP4 from genomic DNA isolated from deGradFP flies (Caussinus et al., 2011) using the primers Forward: AGTGGCGGTGGGCCAAAAAAGAAAAGAAAAAGTCCGCGGCAAATGGATCAAGTCCAACTGGTGG and Reverse: ATGTCACACCACAGAAGTAAGGTTCCTTCACAAAGATCCTTAGCTGGAGACGGTGACCTG. The resulting PCR product was cloned into the pUAST-mCherry-Dam vector (Southall et al., 2013) with XhoI and XbaI sites using Gibson Assembly.

pUAST-attB-hbn was generated by PCR amplifying the *hbn* coding sequence from an embryonic cDNA library using the primers Forward: AGATGAATTCATGATGACCACGACGACCTCG and Reverse: ATGACTCGAGTCAGTCCTCGCCCTTGGTG. The resulting PCR product was cloned into the pUAST-attB vector (Bischof et al., 2007) with EcoRI and XhoI sites.

pUAST-attB-scro was generated by PCR amplifying the *scro-RA* coding sequence from an embryonic cDNA library using the primers Forward: TACCAGGAATTCATGTCATCGCACGGCCTTGCTTAC and Reverse: TAGTATGCGGCCGCTTACCATGCCCGGCCTTGTAAGGG. The resulting PCR product was cloned into the pUAST-attB vector (Bischof et al., 2007) with EcoRI and NotI sites.

#### Short hairpin RNAi generation

Short hairpin *scro* RNAi constructs, pWALIUM20-*scro*-shRNA^5UTR^ (targets *scro* 5UTR) and pWALIUM20*-scro*-shRNA^CDS^ (targets *scro* CDS), were generated by annealing 10 μM concentration of the following oligonucleotide pairs at 98 °C for 5 minutes in annealing buffer (10 mM Tris pH 7.5, 0.1 M NaCl, 1 mM EDTA) and then leaving the mixture to cool at room temperature. The resulting products were cloned into the pWALIUM20 vector (Perkins et al., 2015) with EcoRI and Nhe1 sites.

*hbn*-shRNA^CDS2^ Forward ctagcagtGTTCGAAGATCTCGCACAACAtagttatattcaagcataTGTTGTGCGAGATCTTCGAACgcg and *hbn*-shRNA^CDS2^ Reverse aattcgcGTTCGAAGATCTCGCACAACAtatgcttgaatataactaTGTTGTGCGAGATCTTCGAACactg; *hbn*-shRNA^3UTR^ Forward ctagcagtGATTCGTTATGTACATATATAtagttatattcaagcataTATATATGTACATAACGAATCgcg and *hbn*-shRNA^3UTR^ Reverse aattcgcGATTCGTTATGTACATATATAtatgcttgaatataactaTATATATGTACATAACGAATCactg; *scro*-shRNA^5UTR^ Forward ctagcagtAGTGGATATTCATAATAAATTtagttatattcaagcataAATTTATTATGAATATCCACTgcg and *scro*-shRNA^5UTR^ Reverse aattcgcAGTGGATATTCATAATAAATTtatgcttgaatataacta AATTTATTATGAATATCCACTactg; *scro*-shRNA^CDS^ Forward ctagcagtGCCCAGGTGTACAGACCTATTtagttatattcaagcataAATAGGTCTGTACACCTGGGCgcg and *scro*-shRNA^CDS^ Reverse

aattcgcGCCCAGGTGTACAGACCTATTtatgcttgaatataactaAATAGGTCTGTACACCTGGGCactg. For all expression and RNAi constructs, transgenic flies were generated by germline injection into embryos carrying *y*, *v*, *nos*-phiC integrase; *attP40*; + (BDSC 25709).

#### NanoDam experimental design

To perform Cph NanoDam, a Cph::YFP; UAS-*NanoDam* line was crossed to Cph::YFP; *wor*-GAL4. As a control, UAS-*NanoDam* was crossed to *wor*-GAL4. *wor*-GAL4 is expressed in neuroblasts from approximately stage 10. Embryos were collected at 25 °C and harvested at stage 16. 10-15 μl of embryos were harvested for DNA purification.

For INP NanoDam, flies carrying UAS-*NanoDam*, *tub*-GAL80^ts^ and *D*-GAL4 were crossed to *w*^*1118*^ (control), D-GFP, Grh-GFP or Ey-GFP. For optic lobe NanoDam, flies carrying UAS-*NanoDam*, *tub*-GAL80^ts^ and *ogre*-GAL4 were crossed to *w*^*1118*^, D-GFP, or Ey-GFP. Temporal restriction of NanoDam expression was achieved using GAL80^ts^, a temperature-sensitive negative regulator of GAL4 (Matsumoto et al., 1978; McGuire et al., 2003). Embryos were collected on yeasted apple juice plates at 25 °C and then transferred at 18 °C. Newly hatched larvae were transferred to yeasted food plates and raised at 18 °C for 6 days before shifting to 29 °C for 14 hours. Brains were dissected from wandering third instar larvae in PBS and then transferred to ice-cold PBS. Genomic DNA was extracted from approximately 50 brains per sample.

#### NanoDam sample processing

NanoDam samples were processed using the DamID-seq protocol as described previously (Marshall et al., 2016). DNA was extracted from dissected tissue and methylated fragments were isolated with DpnI and DpnII digestion. Genomic fragments were then amplified by PCR and sonicated in order to generate libraries appropriate for sequencing. Sequencing was performed as single end 50 bp reads generated by an Illumina HiSeq 1500 at the Gurdon Institute NGS Core Facility. Details of biological replicates performed can be found in Figure S2.

#### Single-cell sequencing sample preparation

Embryos of the genotypes *w*; 10xUAS-*IVS-mCD8-RFP*; *D*-GAL4 or *w*; 10xUAS-*IVS-mCD8-RFP*/D-GFP; *D*-GAL4/*+* were collected on yeasted apple juice plates at 25 °C. Newly hatched larvae were transferred to yeasted food plates and reared at 25 °C until wandering third instar stage. Sample preparation prior to FACS was performed as Harzer et al. (2013), but with the use of PBS in place of Schneider’s medium. In brief, larvae were washed in 70 % EtOH/PBS for 1 minute before dissection. Brains from each genotype were then dissected in PBS for one replicate each, transferred to 1.5 ml low-binding tubes with ice-cold Rinaldini solution and then rinsed twice with ice-cold Rinaldini solution. Brains were incubated for 1 hour at 30 °C in dissociation solution (Schneider’s Insect Medium with 1 mg/ml Collagenase I and 1 mg/ml Papain) then rinsed with ice-cold Rinaldini solution, followed by sterile filtered 0.03 % BSA/PBS. The solution was pipetted up and down to dissociate the brains and the resulting cell suspension was passed through a 10 μm mesh filter into a 5 ml FACS tube. SYTOX Blue Dead Cell Stain (Invitrogen) was added to the cell suspension before proceeding to FACS.

FACS was performed using the SH800Z Cell Sorter (Sony) at 4 °C. Single cells were sorted into PBS with 0.03 % Bovine Serum Albumin to prevent clumping. Live, single cells were sorted based on size, and fluorescence intensities of RFP and SYTOX Blue Dead Cell Stain. Gates for FACS were established using a negative control to adjust for autofluorescence and a positive control for SYTOX Blue Dead Cell Stain using dissociated cells incubated at 65 °C for 15 minutes.

Single cell RNA sequencing libraries were generated using 10X Chromium (v2 chemistry kit, 10X Genomics) and sequenced on an Illumina HiSeq 4000 at the Genomics Core Facility, Cancer Research UK Cambridge Institute.

#### Sample fixation and immunostaining

Larval brains were dissected in PBS and fixed on a shaker for 20 minutes in 4 % formaldehyde/PBS. Fixed brains were washed well with PBS containing 0.3 % Triton-X (PBTx) before immunostaining and then for at least 15 minutes in 10 % normal goat serum/PBS. Samples were incubated overnight at 4 °C with primary antibodies diluted in 0.3 % PBTx, washed well with 0.3 % PBTx, then incubated overnight at 4 °C with secondary antibodies diluted in 0.3 % PBTx. Samples were washed well with 0.3 % PBTx then mounted in Vectashield (Vector laboratories) for imaging.

The following primary antisera were used: guinea pig anti-D 1:200 (a gift from Alex Gould), guinea pig anti-Dpn 1:5,000 (Caygill and Brand, 2017), rat anti-Dpn 1:100 (abcam, 11D1BC7, ab195173), rabbit anti-Ey (1:300) (a gift from Uwe Walldorf), chicken anti-GFP 1:2,000 (abcam, ab13970), rat anti-Grh 1:1000 (Baumgardt et al., 2009), rabbit anti-Hbn (1:200) (a gift from Uwe Walldorf). Secondary antibodies conjugated Alexa Fluor dyes (Life Technologies) or DyLight-405 1:200 (Jackson Laboratories) were used to detect primary antibodies.

#### *In situ* hybridisation chain reaction (HCR)

To perform HCR for *scro* mRNA, custom probes designed against the *scro* coding sequence, buffers and fluorophore-labelled amplification hairpins were sourced from Molecular Instruments (Choi et al., 2018). The HCR protocol was adapted for use on third instar *Drosophila* larval brains (Choi et al., 2016). In brief, larval brains were fixed on a shaker at room temperature for 20 minutes in 4 % formaldehyde/PBS. Fixed brains were washed well with PBS and then incubated in probe hybridisation buffer (PHB) (Molecular Instruments) at 37 °C for 10 minutes. PHB was removed from the samples and replaced with pre-warmed probe mix (0.8 μl of probe added to 200 μl PHB). Samples were incubated in probe mix overnight at 37 °C. The following day, samples were washed well with probe wash buffer (PWB) (Molecular Instruments) at 37 °C and then washed with 5XSSC containing 0.1 % Triton-X (5X SSCTx) at room temperature. Samples were incubated in amplification buffer (AB) (Molecular Instruments) at room temperature for at least 10 minutes. AB was removed and replaced with 100 μl AB containing 2 μl each fluorophore-labelled amplification hairpin1 and hairpin2 (Molecular Instruments). Samples were then incubated overnight in the dark at room temperature. Note that hairpins 1 and 2 were heated separately at 95 °C for 1.5 minutes and cooled at room temperature for 30 minutes in the dark before use. The next day, samples were washed well with 5X SSCTx before proceeding to immunostaining processing for antibody co-staining. Amplification hairpins were labelled with Alexa Fluor 488 or 647. HCR samples were mounted in SlowFade Gold antifade reagent (Invitrogen) for imaging.

#### Image acquisition

Fluorescent images were acquired using a Leica SP8 confocal microscope. Images were analyses using Fiji (Schindelin et al., 2012), which was also used to adjust brightness and contrast in images. Adobe Illustrator was used to compile figures.

### Quantification and statistical analysis

#### NanoDam data processing

The quality of all ^∗^.fastq-files was validated by using FastQC (v0.11.5). Data processing was performed with a wrapper script to automate and parallelize the application of the damidseq_pipeline (Marshall and Brand, 2015) with the slurm workload manager (v15.08.13). All ^∗^.fastq.gz-files were mapped with bowtie2 (v2.3.4.1) to the *Drosophila* dm6 genome assembly and all reads were assigned to bins defined by consecutive GATC sites throughout the genome. For NanoDam of each transcription factor, all replicates (4 D-GFP replicates, 5 Grh-GFP replicates, and 4 Ey-GFP replicates) were normalised individually to all control replicates (8 *w*^*1118*^ replicates) followed by quantile normalisation of all pairwise comparisons to each other. Binding profiles for each transcription factor were generated by averaging the binding intensities across all normalised comparisons per GATC-bin. The averaged logarithmic binding intensities were subsequently backtransformed and bedGraphToBigWig (v4) was used to generate ^∗^.bw-files for visualisation of the binding profiles in IGV (Robinson et al., 2011). Broad peaks were called with MACS2 (v2.1.2.) on ^∗^.bam-files derived from the damidseq_pipeline for all NanoDam/control pairwise combinations. Overlapping peak regions were merged with bedtools (v2.26.0) into consensus peaks. Consensus peaks were filtered by false discovery rate (i.e., FDR<10^-25^) and by occurrence in more than 50 % across all pairwise combinations.

For genome-wide correlation analysis of individual libraries, bamCoverage (v3.0.2) was used to aggregate reads from ^∗^.bam-files of individual samples after extension to 150 bp across consecutive 500 bp-bins. Pearson correlation coefficients were calculated with the R stats (v3.6.1) package between these NanoDam, TaDa, NanoDam only and TaDa only libraries or for comparison of normalized, pairwise replicates. Complexity and fingerprint analysis were conducted with preseq (v2.0.0) and custom R scripts adapting plotFingerprint’s algorithm (v3.0.2). Enrichment of binding intensities on peaksets and ROC-like curves were calculated with custom R scripts employing the R rtracklayer package (v1.46.0).

Binding intensities for all GATC-bins overlapping with individual peaks were averaged and normalised for the length of the peak. Intensities for GATC-bins intersecting with the peak borders were weighted depending on the overlap of the respective bin with the peak. The resulting intensities per peak and genotype were converted into z-scores and clustered with the ‘kmeans’ function of the R stats package (v3.6.1). The optimal number of clusters was determined by using the factoextra (v1.0.5), clValid (v0.6-6) and mclust (v5.4.5) packages in R. All considered clustering approaches were evaluated by calculating silhouettes with the cluster (v2.0.7-1) package.

Peaks were assigned to the closest transcriptional start site of protein-coding genes according to ensembl annotations with bedtools (dm6, biomaRt v2.38.0). To identify genes encoding transcription factors the resulting genes were intersected with the curated list of supported *Drosophila* transcription factors from FlyTF.org (https://www.mrc-lmb.cam.ac.uk/genomes/FlyTF/old_index.html).

R markdowns outlining all analytical workflows as well as the suite of Python3 scripts for automated DamIDseq analysis are available at https://github.com/AHBrand-Lab/NanoDam_analysis. Raw and processed data can be acquired at NCBI GEO under the accession number GSE190210.

#### Single-cell sequencing data processing

The acquired single cell RNA sequencing files were mapped and count matrices derived from Cell Ranger (v2.2.1). The required reference transcriptome was build from ^∗^.gtf- and ^∗^.fa-files for the Ensembl dm6 genome assembly. The Dichaete-BAC used to generate the D-GFP (BL66758) was in silico cloned and custom made ^∗^.gtf- and ^∗^.fa-files of the resulting plasmid were combined with the *Drosophila* transcriptome to generate a separate reference for validation of D-GFP expression as a means to quality check the sample preparation. Replicates (with and without D-GFP) were found to be very similar when the two samples were integrated. Data for both single cell data sets were separately normalized and scaled with the Seurat R package (v2.3.4) prior to their integration via canonical correlation analysis (Butler et al., 2018; Stuart et al., 2019). The number of screened and chosen genes (i.e., ‘num.possible.genes’ and ‘num.genes’) while constructing the metagene as well as the number of aligned dimensions were optimized during the subspace alignment. After excluding tracheal clusters (i.e., 5 and 6), significantly differentially expressed genes for all clusters were identified by using the ‘FindMarkers’ command (i.e., “Wilcoxon rank sum test”). To identify transcription factor genes, the list was intersected with the aforementioned list of supported *Drosophila* transcription factors from FlyTF.org.

The entire analysis is outlined in the ‘scRNAseq_analysis.Rmd’ R markdown (https://github.com/AHBrand-Lab/NanoDam_analysis). Raw ^∗^.fastq.gz files and data processed by Cell Ranger (v2.2.1) are deposited at NCBI GEO under the accession number GSE190210.

#### Image analysis and quantification

GraphPad Prism 8 for Mac OS X (www.graphpad.com) was used for statistical analyses. Following normality tests, Mann-Whitney U tests were used to assess the statistical significance between two genotypes and Kruskal-Wallis tests were used when experiments contained more than two genotypes. N numbers for each experiment are found in the figure legends. Error bars indicate the standard deviation (SD) and is also noted in the figure legends.

## Data Availability

Single-cell RNA-seq and NanoDam data have been deposited at GEO and are publicly available as of the date of publication. Any additional information required to reanalyze the data reported in this paper is available from the lead contact upon request. Accession numbers are listed in the key resources table. All original code has been deposited at GitHub and publicly available of the date of publication. DOIs are listed in the key resources table. Microscopy data reported in this paper will be shared by the lead contact upon request.

## Supplemental information

Document S1. Figures S1–S7 and Tables S1–S3

Document S2. Article plus supplemental information
