# Supplementary material for: NanoDam identifies Homeobrain (ARX) and Scarecrow (NKX2.1) as conserved temporal factors in the Drosophila central brain and visual system
Source: Dev Cell. 2022 May 9;57(9):1193–1207.e7. doi: 10.1016/j.devcel.2022.04.008 (PMC9616798; doi:10.1016/j.devcel.2022.04.008)
Supplement: Document S1. Figures S1–S7 and Tables S1–S3 [file mmc1.pdf]

Developmental Cell, Volume *57*

Supplemental information

NanoDam identifies Homeobrain (ARX) and Scarecrow (NKX2.1) as conserved temporal factors in the *Drosophila* central brain and visual system

Jocelyn L.Y. Tang, Anna E. Hakes, Robert Krautz, Takumi Suzuki, Esteban G. Contreras, Paul M. Fox, and Andrea H. Brand

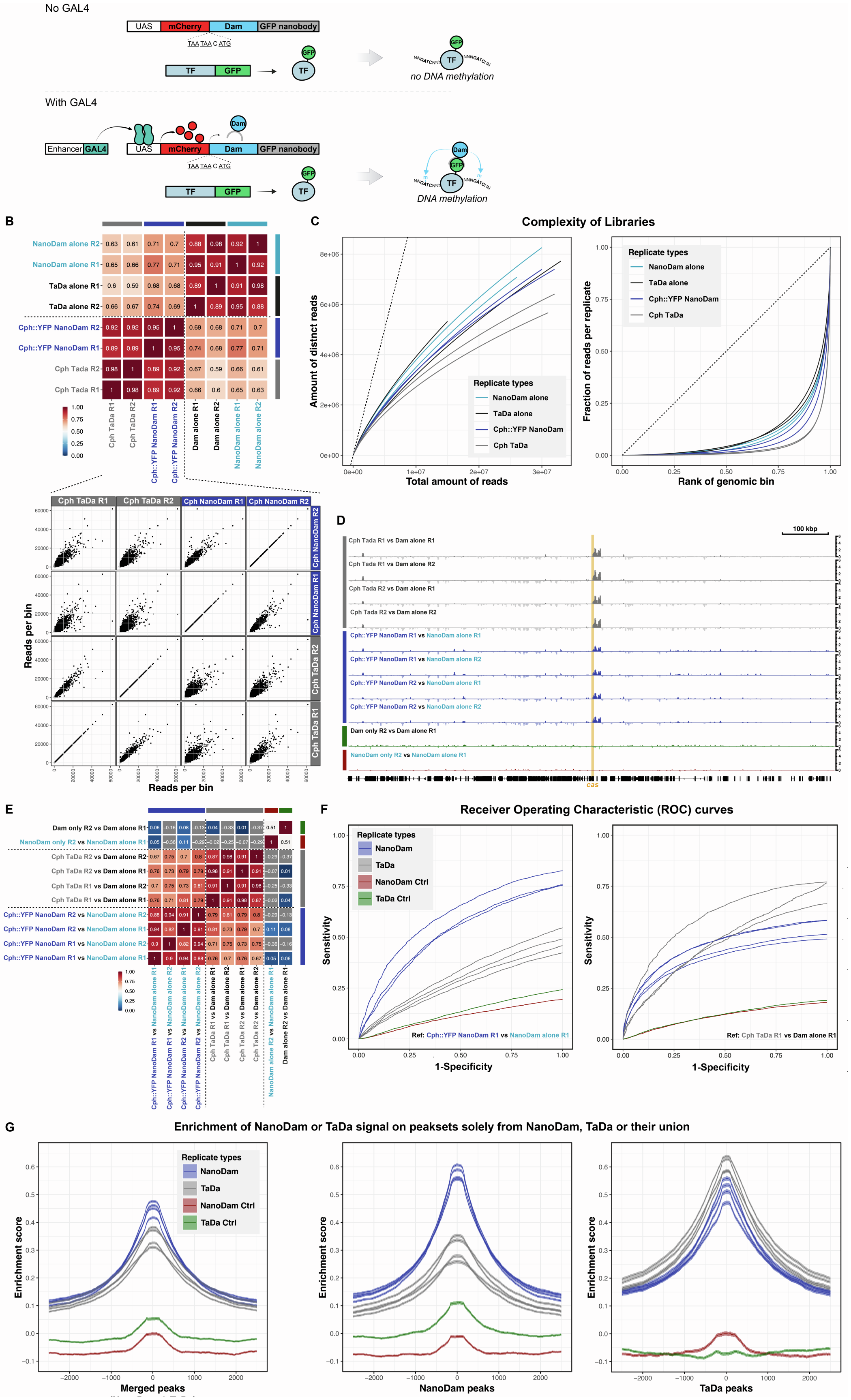

No GAL4
UAS
mCherry
Dam
GFP nanobody
TAA TAA C ATG
TF
GFP
no DNA methylation
With GAL4
Enhancer
GAL4
DNA methylation
B
NanoDam alone R2
NanoDam alone R1
TaDa alone R1
TaDa alone R2
Cph::YFP NanoDam R2
Cph::YFP NanoDam R1
Cph Tada R2
Cph Tada R1
Cph TaDa R1
Cph TaDa R2
Cph::YFP NanoDam R1
Cph::YFP NanoDam R2
Dam alone R1
Dam alone R2
Cph NanoDam R1
Cph NanoDam R2
Reads per bin
C
Complexity of Libraries
Amount of distinct reads
Total amount of reads
Replicate types
NanoDam alone
TaDa alone
Cph::YFP NanoDam
Cph TaDa
Fraction of reads per replicate
Rank of genomic bin
D
100 kbp
Cph Tada R1 vs Dam alone R1
Cph Tada R1 vs Dam alone R2
Cph Tada R2 vs Dam alone R1
Cph Tada R2 vs Dam alone R2
Cph::YFP NanoDam R1 vs NanoDam alone R1
Cph::YFP NanoDam R1 vs NanoDam alone R2
Cph::YFP NanoDam R2 vs NanoDam alone R1
Cph::YFP NanoDam R2 vs NanoDam alone R2
Dam only R2 vs Dam alone R1
NanoDam only R2 vs NanoDam alone R1
cas
E
Dam only R2 vs Dam alone R1
NanoDam only R2 vs NanoDam alone R1
Cph TaDa R2 vs Dam alone R2
Cph TaDa R2 vs Dam alone R1
Cph TaDa R1 vs Dam alone R2
Cph TaDa R1 vs Dam alone R1
Cph::YFP NanoDam R2 vs NanoDam alone R2
Cph::YFP NanoDam R2 vs NanoDam alone R1
Cph::YFP NanoDam R1 vs NanoDam alone R2
Cph::YFP NanoDam R1 vs NanoDam alone R1
F
Receiver Operating Characteristic (ROC) curves
Replicate types
NanoDam
TaDa
NanoDam Ctrl
TaDa Ctrl
Sensitivity
1-Specificity
Ref: Cph::YFP NanoDam R1 vs NanoDam alone R1
Ref: Cph TaDa R1 vs Dam alone R1
G
Enrichment of NanoDam or TaDa signal on peaksets solely from NanoDam, TaDa or their union
Enrichment score
Merged peaks
NanoDam peaks
TaDa peaks

Figure S1: NanoDam methodology and comparisons with TaDa, Related to Figure 1.

(**A**) Schematic representation of the NanoDam method. The NanoDam construct (the GFP nanobody fused to the C-terminus of Dam methylase is under the control of the GAL4 UAS, allowing its expression to be activated in a cell-type-specific manner by GAL4. Translation of the NanoDam construct is greatly reduced by the addition of an upstream ORF (mCherry) followed by two stop codons, which prevents NanoDam expression in uninduced cells and nonspecific methylation (caused by high NanoDam levels) in induced cells.

(**B**) Correlation matrix with Pearson correlation coefficients for genome-wide, paired comparisons between individual replicates (R1 or R2) of Cph::NanoDam, Cph TaDa, NanoDam only and TaDa only.

(**C**) Complexity plot (left) illustrating the number of distinct reads as a function of the total number of reads for each library across all replicate types (Cph::NanoDam, Cph TaDa, NanoDam only and TaDa only). Fingerprint plot (right) representing cumulative read coverage as the fraction of reads for each library for consecutively added (ranked) genomic bins (500 bp) across all replicates.

(**D**) Binding profiles of Cph at the *cas* locus for pairwise comparisons of Cph::YFP NanoDam or Cph TaDa replicates normalised to their respective NanoDam only or Dam only controls. Data is represented as log2-fold enrichment between replicates.

(**E**) Correlation matrix of genome-wide correlation analyses showing the Pearson correlation coefficient of Cph::YFP NanoDam and Cph TaDa replicates normalised over controls.

(**F**) ROC-like curves (Receiver Operating Characteristic) quantifying the amount of overlap between peaksets of normalised replicates compared to either a NanoDam (left: Cph::YFP NanoDam R1 vs NanoDam R1) or a TaDa reference (right: Cph TaDa R1 vs TaDa only R1). Sensitivity represents the fraction of peaks in one replicate also present in the reference peaks (Sensitivity is never 100% as overlap between any two peaksets is never perfect). 1-Specificity represents the fraction of peaks in one replicate that is not present in the reference peaks.

(**G**) Enrichment analysis for binding intensitites for individual, normalised Cph::YFP NanoDam and Cph TaDa replicates across peaksets derived from the union of both, NanoDam and TaDa (left), NanoDam alone (middle) or TaDa alone (right).

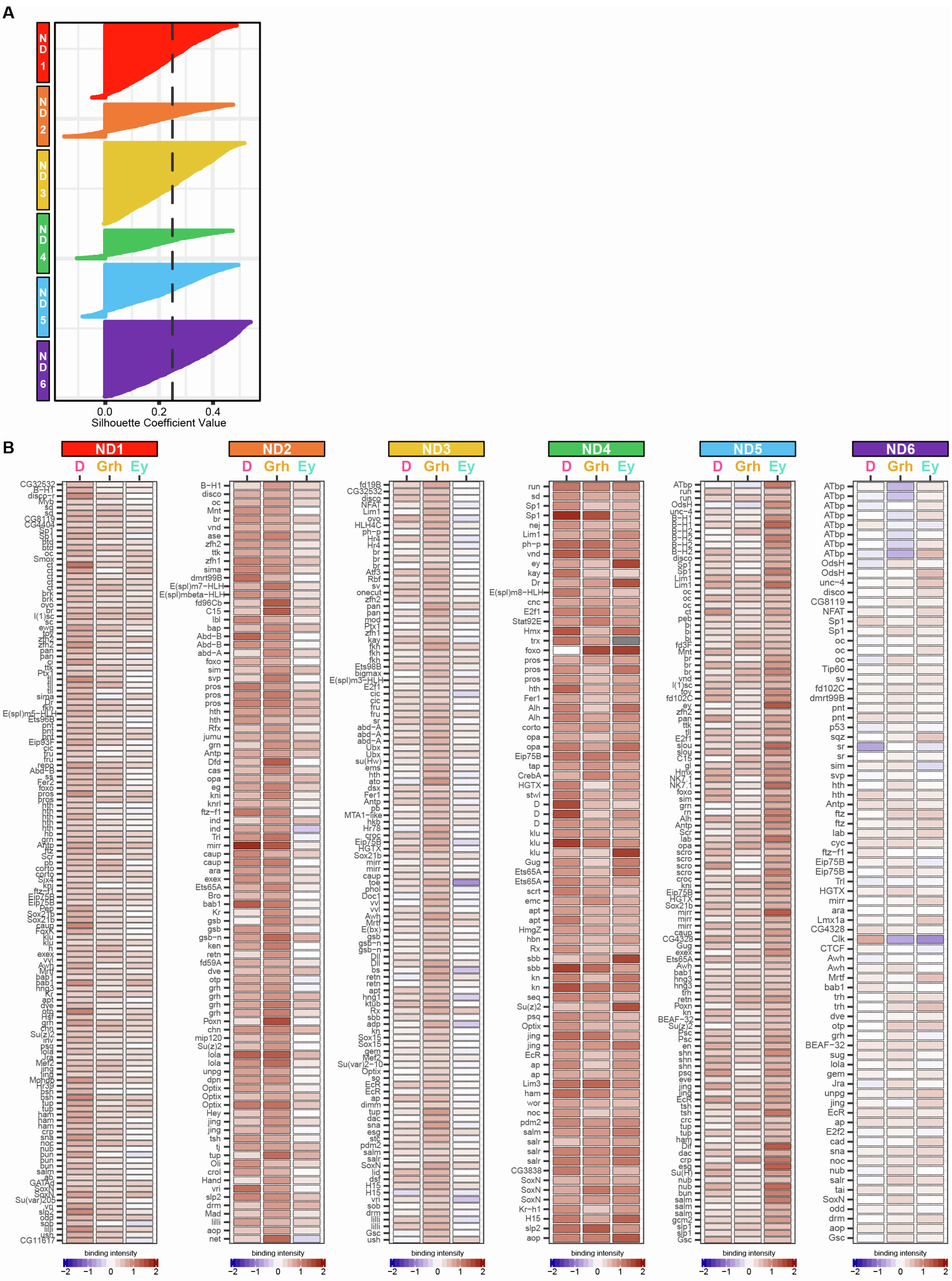
A
ND1
ND2
ND3
ND4
ND5
ND6
0.0
0.2
0.4
Silhouette Coefficient Value
B
ND1
ND2
ND3
ND4
ND5
ND6
D
Grh
Ey
binding intensity
−2
−1
0
1
2

Figure S2: NanoDam of D, Grh and Ey in INPS, Related to Figure 2.

(**A**) Silhouette plot for all clustered peaks provides an indicator for the robustness of the assignment to their respective clusters. Dashed line represents the average silhouette length across all clusters.

(**B**) For each cluster (ND1-6), peaks were annotated to the nearest genes on the linear genome and then subsetted for transcription factors to generate the shown heatmaps. NanoDam binding intensities for D, Grh or Ey are shown as z-scores for individual peaks. Each box represents the intensity of a single peak.

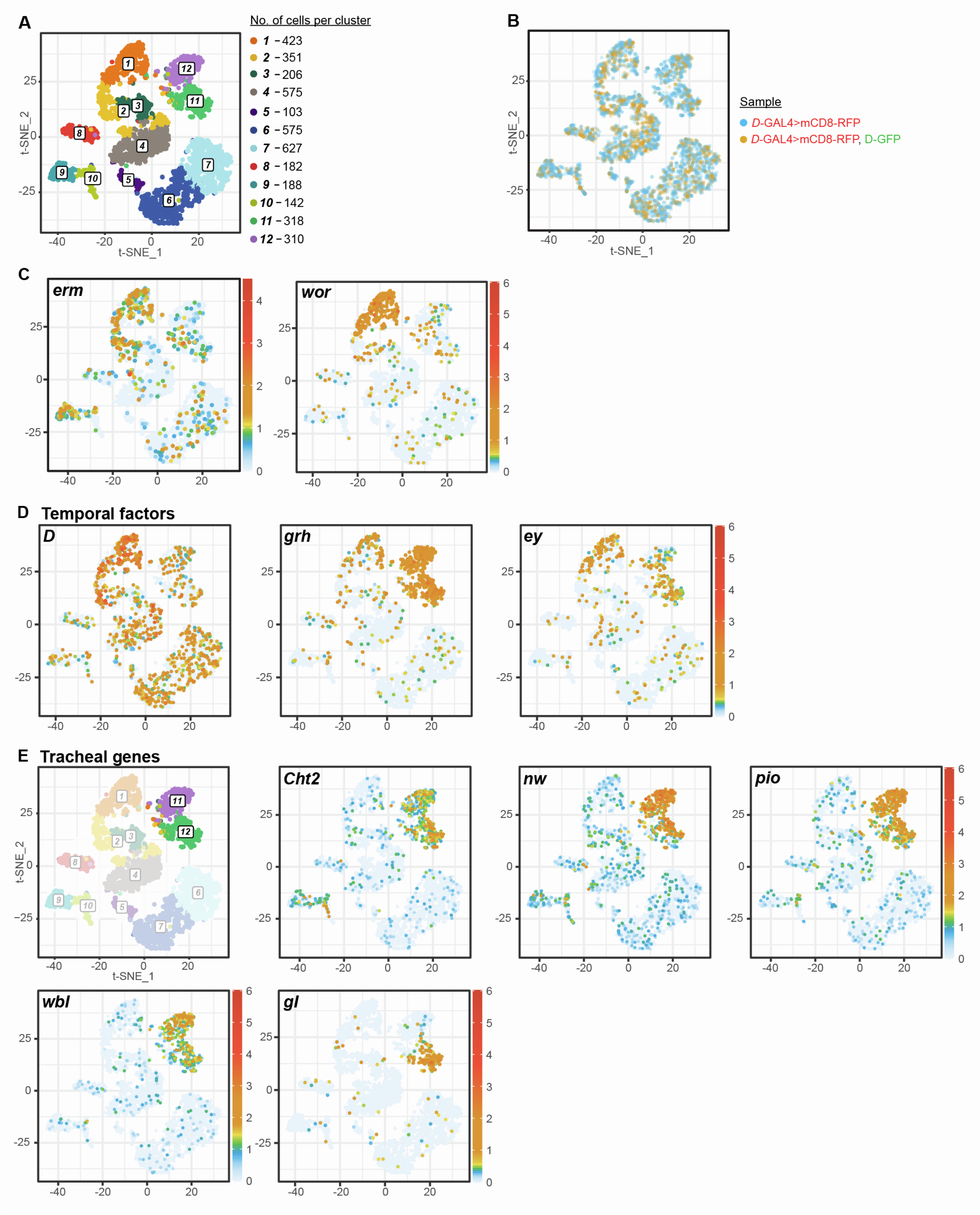

A
No. of cells per cluster
1 – 423
2 – 351
3 – 206
4 – 575
5 – 103
6 – 575
7 – 627
8 – 182
9 – 188
10 – 142
11 – 318
12 – 310
t-SNE_1
t-SNE_2
B
Sample
D-GAL4>mCD8-RFP
D-GAL4>mCD8-RFP, D-GFP
C
erm
wor
D Temporal factors
D
grh
ey
E Tracheal genes
Cht2
nw
pio
wbl
gl

Figure S3: Assigning cell identities using known marker genes, Related to Figure 3.

(**A**) t-SNE visualisation of 4,086 cells derived from both replicates and coloured by cluster assignment. Numbers in the legend indicate the amount of cells per cluster.
(**B**) t-SNE plot showing the cells contributed by the two replicates to the clusters.
(**C**) t-SNE visualisation showing expression of INP marker genes *earmuff* (*erm*) and *worniu* (*wor*).
(**D**) t-SNE plots coloured by expression of the temporal factors in INPs: *Dichaete (D)*, *grainyhead* (*grh*) and *eyeless (ey).*
(**E**) Clusters 11 and 12 correspond to presumed trachea due to strong enrichment of *Chitinase 2* (*Cht2*), *narrow* (*nw*) *piopio* (*pio*) (Affolter and Caussinus, 2008), *windbeutel* (*wbl*), *glass* (*gl*).

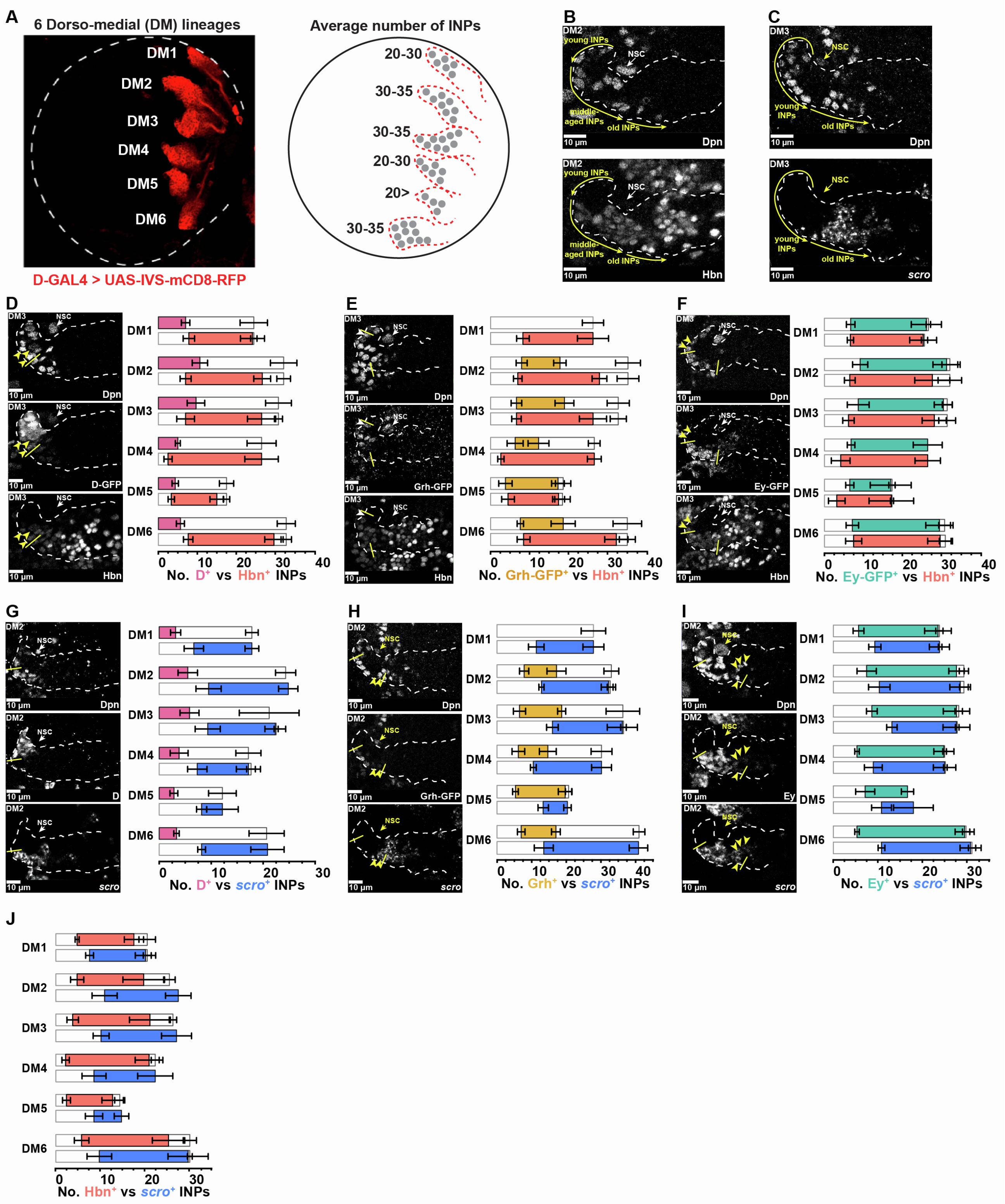

A
6 Dorso-medial (DM) lineages
DM1
DM2
DM3
DM4
DM5
DM6
D-GAL4 > UAS-IVS-mCD8-RFP
Average number of INPs
20-30
30-35
30-35
20-30
20>
30-35
B
DM2
young INPs
NSC
middle-aged INPs
old INPs
10 µm
Dpn
Hbn
C
DM3
NSC
young INPs
old INPs
10 µm
Dpn
scro
D
DM3
NSC
Dpn
D-GFP
Hbn
No. D+ vs Hbn+ INPs
E
Grh-GFP
No. Grh-GFP+ vs Hbn+ INPs
F
Ey-GFP
No. Ey-GFP+ vs Hbn+ INPs
G
DM2
D
scro
No. D+ vs scro+ INPs
H
Grh-GFP
No. Grh+ vs scro+ INPs
I
Ey
No. Ey+ vs scro+ INPs
J
DM1
DM2
DM3
DM4
DM5
DM6
0
10
20
30
40
No. Hbn+ vs scro+ INPs

Figure S4: Overlap of Homeobrain and *scarecrow* with D, Grh and Ey in INPs in DM1-6 lineages, Related to Figure 4.

(**A**) Schematic showing the 6 dorso-medial (DM) lineages and average number of INPs per lineage.

(**B**) Grayscale images of Fig. 4A' showing Dpn and Hbn expression.

(**C**) Grayscale images of Fig. 4H' showing Dpn and *scro* expression.

(**D**) Grayscale images of Fig. 4C and quantification of D/Hbn overlap.

(**E**) Grayscale images of Fig. 4D and quantification of Grh/Hbn overlap.

(**F**) Grayscale images of Fig. 4E and quantification of Ey/Hbn overlap.

(**G**) Grayscale images of Fig. 4I and quantification of D/*scro* overlap.

(**H**) Grayscale images of Fig. 4J and quantification of Grh/*scro* overlap.

(**I**) Grayscale images of Fig. 4K and quantification of Ey/*scro* overlap.

(**J**) Quantification of Hbn/*scro* overlap.

Single section confocal images. White dotted lines indicate *D*-GAL4>*mCD8-RFP*. Brains were dissected at wandering third instar stage. Error bars represent standard deviation of each window. *n* = 6 brain lobes for all conditions.

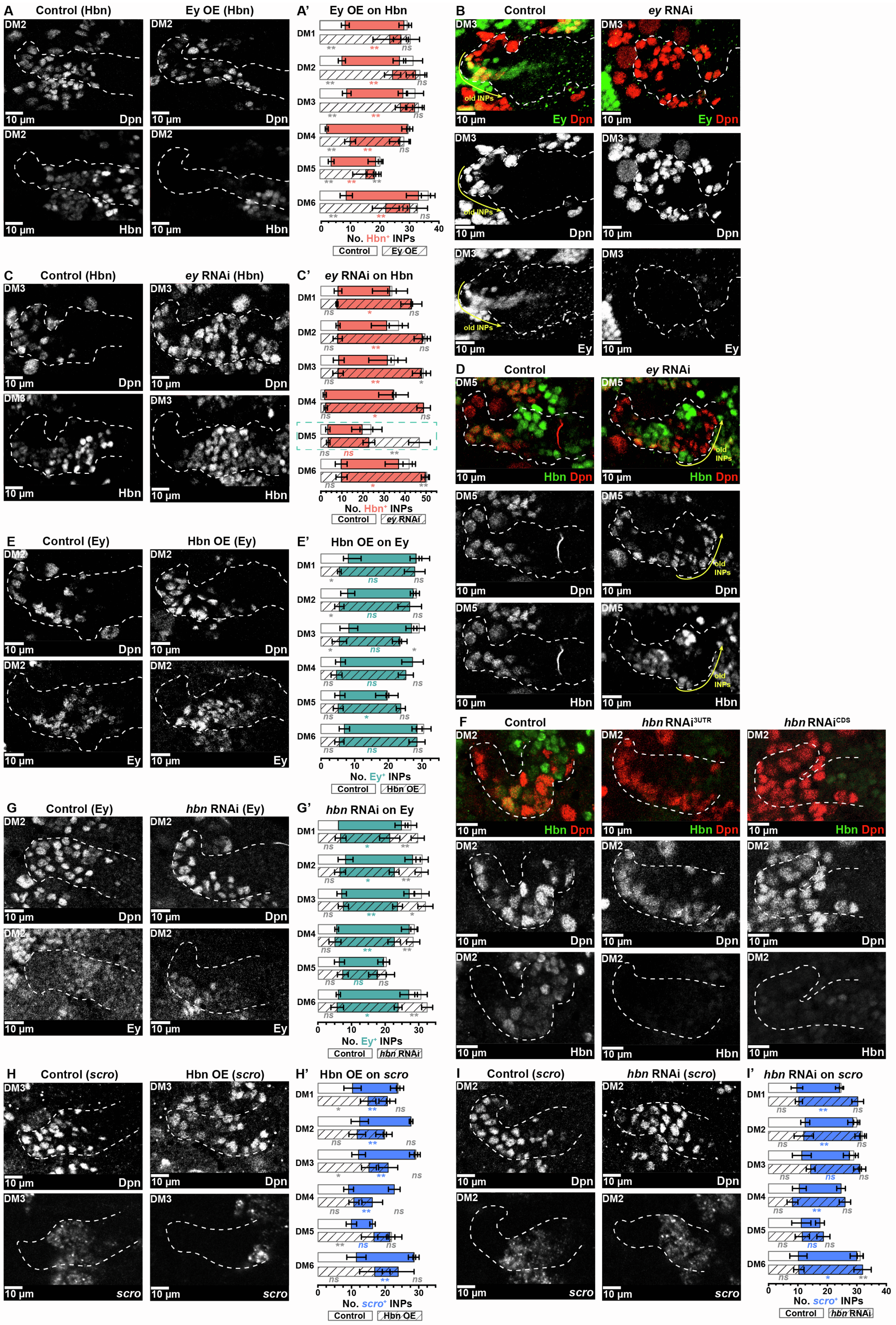
A Control (Hbn)
Ey OE (Hbn)
A' Ey OE on Hbn
No. Hbn+ INPs
Control
Ey OE
B Control
ey RNAi
old INPs
Ey Dpn
C Control (Hbn)
ey RNAi (Hbn)
C' ey RNAi on Hbn
No. Hbn+ INPs
Control
ey RNAi
D Control
ey RNAi
Hbn Dpn
E Control (Ey)
Hbn OE (Ey)
E' Hbn OE on Ey
No. Ey+ INPs
Control
Hbn OE
F Control
hbn RNAi3UTR
hbn RNAiCDS
G Control (Ey)
hbn RNAi (Ey)
G' hbn RNAi on Ey
No. Ey+ INPs
Control
hbn RNAi
H Control (scro)
Hbn OE (scro)
H' Hbn OE on scro
No. scro+ INPs
Control
Hbn OE
I Control (scro)
hbn RNAi (scro)
I' hbn RNAi on scro
No. scro+ INPs
Control
hbn RNAi
DM1
DM2
DM3
DM4
DM5
DM6
10 µm
Dpn
Hbn
Ey
scro

Figure S5: Regulatory relationships of Hbn, Related to Figure 5.

(**A-A'**) Grayscale images of Fig. 5A of Dpn and Hbn expression. Quantifications of the Hbn temporal window split by DM lineage and significance tested by Mann-Whitney Test.

(**B**) Control (w^{1118}) DM lineages expressed Ey (green) in old INPs (Dpn^{+} (red), yellow arrow). Driving *ey* RNAi resulted in the loss of Ey in INPs. RNAi expression was driven by *D*-GAL4>*mCD8-RFP* in combination with UAS-*Dcr2*. Images are projections over 8 µm in z.

(**C-C'**) Grayscale images of Fig. 5B of Dpn and Hbn expression. Quantifications of the effect of *ey* RNAi on Hbn expression split by DM lineage and significance tested by Mann-Whitney Test.

(**D**) *ey* RNAi extends the Hbn temporal window in all lineages except DM5.

(**E-E'**) Grayscale images of Fig. 5C showing Dpn and Ey expression. Quantifications of the Ey temporal window split by DM lineage and significance tested by Mann-Whitney Test.

(F) Driving *hbn* RNAi^{3UTR} or *hbn* RNAi^{CDS} in INPs resulted in the loss of Hbn (green) indicating effective knockdown. Control is w^{1118}.

(**G-G'**) Grayscale images of Fig. 5D showing Dpn and Ey expression. Quantifications of the Ey temporal window split by DM lineage and significance tested by Mann-Whitney Test.

(**H-H'**) Grayscale images of Fig. 5E showing Dpn and *scro* expression. Quantifications of the *scro* temporal window split by DM lineage and significance tested by Mann-Whitney Test.

(**I-I'**) Grayscale images of Fig. 5F showing Dpn and *scro* expression. Quantifications of the *scro* temporal window split by DM lineage and significance tested by Mann-Whitney Test.

p-values: ns, 0.05< ; *, <0.05 ; **, <0.01; ***, <0.001. Error bars represent standard deviation of each window. Single section confocal images. White dotted lines indicate *D*-GAL4>*mCD8-RFP* expression. Brains were dissected at wandering third instar stage. *n* = 6 brain lobes for all experiments.

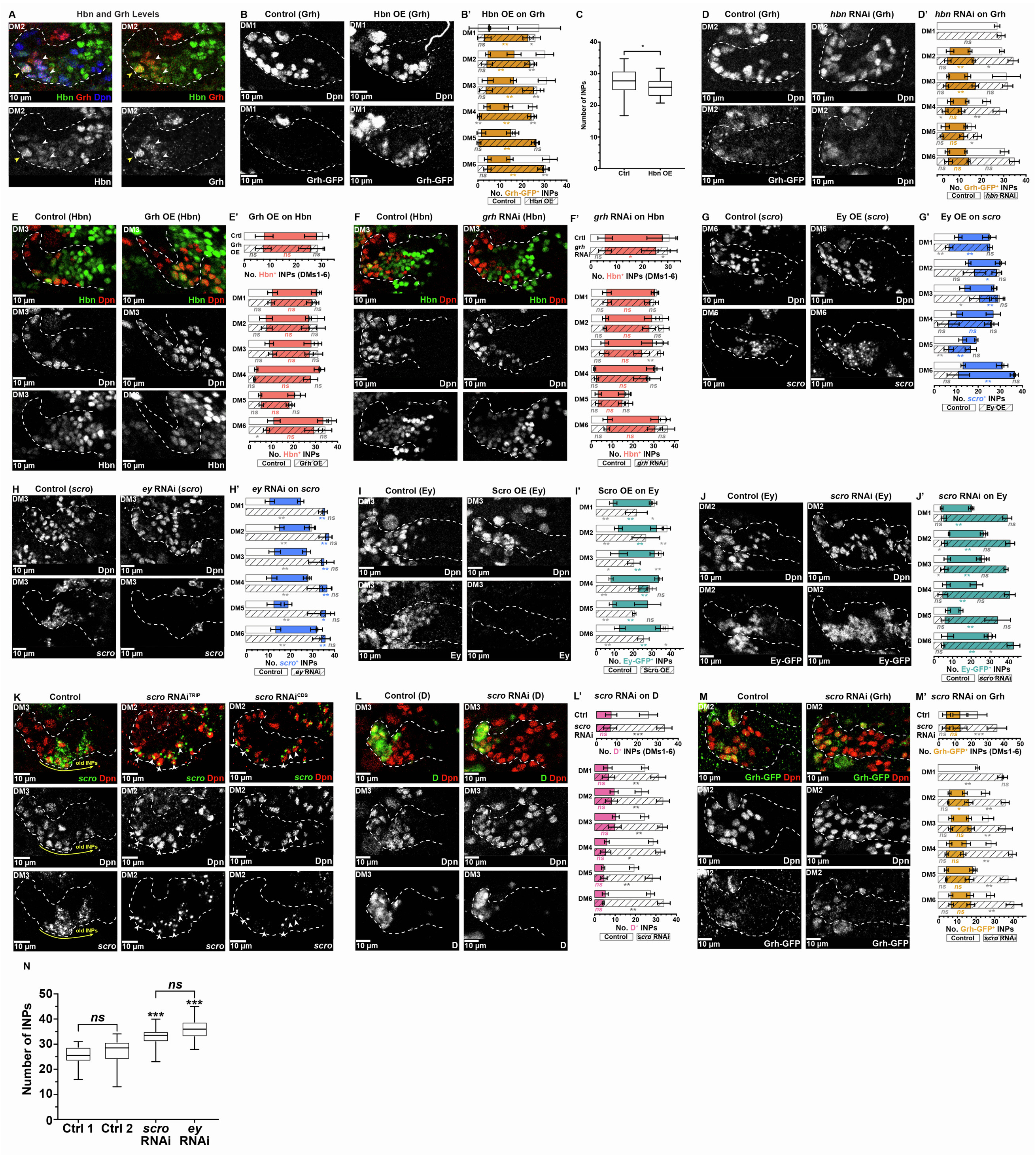
A Hbn and Grh Levels
Hbn Grh Dpn
B Control (Grh)
Hbn OE (Grh)
Dpn
Grh-GFP
B' Hbn OE on Grh
No. Grh-GFP+ INPs
Control
Hbn OE
C
Number of INPs
Ctrl
Hbn OE
D Control (Grh)
hbn RNAi (Grh)
D' hbn RNAi on Grh
Control
hbn RNAi
E Control (Hbn)
Grh OE (Hbn)
E' Grh OE on Hbn
No. Hbn+ INPs (DMs1-6)
No. Hbn+ INPs
Grh OE
F Control (Hbn)
grh RNAi (Hbn)
F' grh RNAi on Hbn
grh RNAi
G Control (scro)
Ey OE (scro)
G' Ey OE on scro
No. scro+ INPs
Ey OE
H Control (scro)
ey RNAi (scro)
H' ey RNAi on scro
ey RNAi
I Control (Ey)
Scro OE (Ey)
I' Scro OE on Ey
No. Ey-GFP+ INPs
Scro OE
J Control (Ey)
scro RNAi (Ey)
J' scro RNAi on Ey
scro RNAi
K Control
scro RNAi[TRiP]
scro RNAi[CDS]
old INPs
L Control (D)
scro RNAi (D)
L' scro RNAi on D
No. D+ INPs (DMs1-6)
No. D+ INPs
M Control
scro RNAi (Grh)
Grh-GFP Dpn
M' scro RNAi on Grh
No. Grh-GFP+ INPs (DMs1-6)
N
Number of INPs
Ctrl 1
Ctrl 2
scro RNAi
ey RNAi
ns
***
10 μm

Figure S6: Regulatory relationships of Hbn and *scro*, Related to Figures 5 and 6.

(**A**) The strength of Hbn and Grh expression levels correlate with each other. Yellow arrows indicate strong Hbn and Grh expression, white arrows indicate weaker expressions in INPs.

(**B-B'**) Grayscale images of Fig. 5G showing Dpn and Grh-GFP expression. Quantification of Hbn overexpression on Grh expression in INPs split by DM lineages and significance tested by Mann-Whitney Test.

(C) Overexpression of Hbn leads to a decrease in INP number. Mann-Whitney Test p=0.041, *.

(**D-D'**) Grayscale images show Dpn and Grh expression separately (Fig. 5H) and quantifications also shown split by DM lineage. Quantification of Hbn overexpression on Grh expression in INPs split by DM lineages and significance tested by Mann-Whitney Test.

(**E-E'**) Misexpression of Grh does not affect Hbn expression. Mann-Whitney Test (averaged across DMs1-6) p=0.73, ns; p=0.09, ns; p=0.083, ns. Grayscale images show Dpn and Hbn expression separately and quantifications also shown split by DM lineage.

(**F-F'**) Loss of Grh does not affect the Hbn window. Mann-Whitney Test (averaged across DMs1-6) p=0.93, ns; p=0.04, *; p=0.03, *. Grayscale images show Dpn and Hbn expression separately and quantifications also shown split by DM lineage.

p-values (unless stated): ns, 0.05< ; *, <0.05 ; **, <0.01; ***, <0.001.

(**G-G'**) Ey misexpression precociously activates *scro* in INPs in all lineages except DM2 and DM3. Grayscale images of Fig. 6A showing Dpn and scro expression separately. Quantifications are also split by DM lineage and significance tested by Mann-Whitney Test.

(**H-H'**) Grayscale images of Fig. 6B showing Dpn and scro expression separately. Quantifications are also split by DM lineage and significance tested by Mann-Whitney Test.

(**I-I'**) Grayscale images of Fig. 6C showing Dpn and Ey expression separately. Quantifications are also split by DM lineage and significance tested by Mann-Whitney

Test.

(**J-J'**) Grayscale images of Fig. 6D showing Dpn and Ey-GFP expression separately. Quantifications are also split by DM lineage and significance tested by Mann-Whitney Test.

(**K**) Expressing a control RNAi (*mCherry* RNAi) did not affect the expression of *scro* (green) in INPs (Dpn^{+}, highlighted by yellow arrow). Driving *scro* RNAi^{TRiP} or *scro* RNAi^{CDS} in INPs resulted in the loss of *scro* mRNA from the cytoplasm of old INPs (arrowheads) indicating effective knockdown. Note that *scro* mRNA can still be detected in the nucleus even when driving *scro* RNAi.

(**L-L'**) *scro* RNAi does not affect D expression in INPs. Control line is *mCherry* RNAi. Mann-Whitney Test p=0.36, ns; p<0.001, ***. Grayscale images show Dpn and D expression separately and quantifications also shown split by DM lineage.

(**M-M'**) *scro* RNAi does not affect Grh expression in INPs. Control line is *mCherry* RNAi. Mann-Whitney Test p=0.26, ns; p=0.41, ns; p<0.001, ***. Grayscale images show Dpn and Grh expression separately and quantifications also shown split by DM lineage.

(N) Knockdown of *scro* or *ey* leads to an increase in the number of INPs. Quantification of the number of INPs in control DM lineages compared to *scro* RNAi or *ey* RNAi lineages. Control 1 is *mCherry* RNAi, Control 2 is w^{1118} and *scro* RNAi is *scro* RNAi^{TRiP}. Kruskal-Wallis Test with Dunn's Test. Ctrl1 vs *scro* RNAi: p<0.001, ***; Ctrl1 vs *ey* RNAi: p<0.001, ***; Ctrl2 vs *scro* RNAi: p<0.001, ***; Ctrl2 vs *ey* RNAi: p<0.001, ***; *scro* RNAi vs ey RNAi: p>0.99, ns

p-values (unless stated): ns, 0.05< ; *, <0.05 ; **, <0.01; ***, <0.001. Error bars represent standard deviation of each window. Single section confocal images. White dotted lines indicate *D*-GAL4>*mCD8-RFP* expression. Brains were dissected at wandering third instar stage. *n* = 6 brain lobes for all experiments.

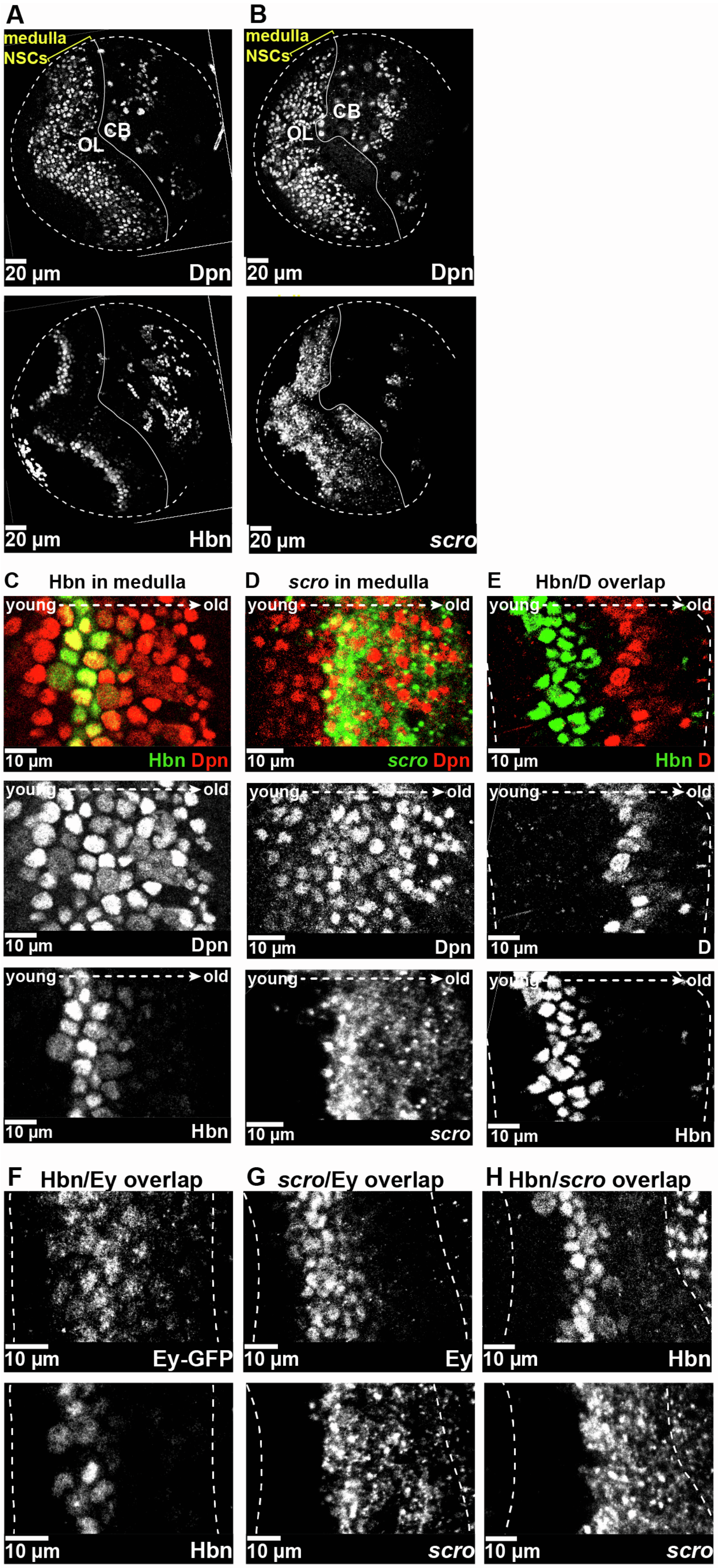

A
medulla
NSCs
OL
CB
20 µm
Dpn
B
medulla
NSCs
CB
OL
20 µm
Dpn
20 µm
Hbn
20 µm
scro
C
Hbn in medulla
young
old
10 µm
Hbn Dpn
10 µm
Dpn
10 µm
Hbn
D
scro in medulla
young
old
10 µm
scro Dpn
10 µm
Dpn
10 µm
scro
E
Hbn/D overlap
young
old
10 µm
Hbn D
10 µm
D
10 µm
Hbn
F
Hbn/Ey overlap
10 µm
Ey-GFP
10 µm
Hbn
G
scro/Ey overlap
10 µm
Ey
10 µm
scro
H
Hbn/scro overlap
10 µm
Hbn
10 µm
scro

Figure S7: Hbn and *scro* as optic lobe temporal factors, Related to Figure 7.

(**A**) Grayscale images of Fig. 7B showing Dpn and Hbn expression separately.

(**B**) Grayscale images of Fig. 7C showing Dpn and *scro* expression separately.

(**C**) Hbn (green) is expressed in a subset of the young OL medulla NSCs (Dpn^+ (red)). Grayscale images show Dpn and Hbn expression separately.

(**D**) *scro* (green) is expressed in the old OL medulla NSCs (Dpn^+). Grayscale images show Dpn and *scro* expression separately.

(**E**) Hbn (green) and D (red) do not overlap in the OL medulla and the Hbn temporal window precedes the D window. Grayscale images show Dpn and D expression separately.

(**F**) Grayscale images of Fig. 7D showing Ey-GFP and Hbn expression separately.

(**G**) Grayscale images of Fig. 7E showing Ey and *scro* expression separately.

(**H**) Grayscale images of Fig. 7F showing Hbn and *scro* expression separately.

Tables

ND1	ND2	ND3	ND4	ND5	ND6
apt<br>*ftz-f1*<br>*grh*<br>*ham*<br>*hng3*<br>*klu*<br>*Myb*<br>*slp2*<br>*sna*<br>*SoxN*	*ase*<br>*cas*<br>*dpn*<br>*ftz-f1*<br>*grh*<br>*jumu*<br>*ken*<br>*opa*<br>*Optix*<br>*slp2*	*apt*<br>*E2f1*<br>*Hr4*<br>*ktub*<br>*Optix*<br>*ph-p*<br>*sna*<br>*SoxN*<br>*su(Hw)*<br>*Su(var)2-10*	*Alh*<br>*apt*<br>*CrebA*<br>*D*<br>*E2f1*<br>*ey*<br>*ham*<br>*hbn*<br>*klu*<br>*opa*<br>*Optix*<br>*ph-p*<br>*run*<br>*slp2*<br>*SoxN*<br>*tap*<br>*wor*	*Alh*<br>*BEAF-32*<br>*E2f1*<br>*ey*<br>*fd102C*<br>*ham*<br>*hng3*<br>*opa*<br>*run*<br>*scro*	*BEAF-32*<br>*E2f2*<br>*fd102C*<br>*ftz-f1*<br>*grh*<br>*sna*<br>*SoxN*

Table S1.

Genes bound by D/Grh/Ey (NanoDam) and expressed in INPs (scRNA-seq), Related to Fig. 3.

GFP Fusion Libraries	No. of lines
Endogenous Tag	
CPTI (Lowe et al., 2014)	616
FlyTrap (Kelso et al., 2004)	1,150
MiMIC RMCE (Nagarkar-Jaiswal et al., 2015)	653
BAC-based insertion tags	
fly-TransgeneOme (Sarov et al., 2016)	895
modERN Resource (Kudron et al., 2018)	552
P[acman] BAC (Venken et al., 2009)	113

Table S2, Related to Fig. 1.

GFP-fusion libraries publicly available.

Figure and Experiment	Genotype
Fig. 5A: Ey misexpression on Hbn	Crtl: *D*-GAL4>UAS-IVS-mCD8-RFP x w^{1118}<br>Ey OE: *D*-GAL4>UAS-IVS-mCD8-RFP x UAS-Ey
Fig. 5B: *ey* knockdown on Hbn	Crtl: *D*-GAL4>UAS-IVS-mCD8-RFP, UAS-Dcr2 x w^{1118}<br>*ey* RNAi: *D*-GAL4>UAS-IVS-mCD8-RFP, UAS-Dcr2 x Ey-RNAi (KK)
Fig. 5C: Hbn misexpression of Ey	Crtl: *D*-GAL4>UAS-IVS-mCD8-RFP x w^{1118}<br>Hbn OE: *D*-GAL4>UAS-IVS-mCD8-RFP x UAS-Hbn
Fig. 5D: *hbn* knockdown on Ey	Crtl: *D*-GAL4>UAS-IVS-mCD8-RFP x w^{1118}<br>*hbn* RNAi: *D*-GAL4>UAS-IVS-mCD8-RFP x UAS- *hbn*-$RNAi^{CDS2}$
Fig. 5E: Hbn misexpression on *scro*	Crtl: *D*-GAL4>UAS-IVS-mCD8-RFP x w^{1118}<br>Hbn OE: *D*-GAL4>UAS-IVS-mCD8-RFP x UAS-Hbn
Fig. 5F: *hbn* knockdown on *scro*	Crtl: *D*-GAL4>UAS-IVS-mCD8-RFP x w^{1118}<br>*hbn* RNAi: *D*-GAL4>UAS-IVS-mCD8-RFP x UAS- *hbn*-$RNAi^{CDS2}$
Fig. 5G: Hbn misexpression on Grh	Crtl: *D*-GAL4>UAS-IVS-mCD8-RFP x w^{1118}<br>Grh OE: *D*-GAL4>UAS-IVS-mCD8-RFP x UAS-Grh
Fig. 5H: *hbn* knockdown on Grh	Crtl: *D*-GAL4>UAS-IVS-mCD8-RFP x w^{1118}<br>*hbn* RNAi: *D*-GAL4>UAS-IVS-mCD8-RFP x UAS- *hbn*-$RNAi^{CDS2}$
Fig. 6A: Ey misexpression on *scro*	Crtl: *D*-GAL4>UAS-IVS-mCD8-RFP x w^{1118}<br>Ey OE: *D*-GAL4>UAS-IVS-mCD8-RFP x UAS-Ey
Fig. 6B: *ey* knockdown on *scro*	Crtl: *D*-GAL4>UAS-IVS-mCD8-RFP, UAS-Dcr2 x w^{1118}<br>*ey* RNAi: *D*-GAL4>UAS-IVS-mCD8-RFP, UAS-Dcr2 x Ey-RNAi (KK)
Fig. 6C: Scro misexpression on Ey	Crtl: *D*-GAL4>UAS-IVS-mCD8-RFP x w^{1118}<br>Scro OE: *D*-GAL4>UAS-IVS-mCD8-RFP x UAS-Scro
Fig. 6D: *scro* knockdown on Ey	Crtl: *D*-GAL4>UAS-IVS-mCD8-RFP x UAS-RNAi-mCherry

	scro RNAi: *D*-GAL4>UAS-IVS-mCD8-RFP x UAS-*scro*-RNAi (TRiP)
Fig. 7G: *scro* knockdown on Ey	Crtl: hsFLP^{122}; Ay-GAL4, UAS-GFP x w^{1118}<br>*scro* RNAi: hsFLP^{122}. Ay-GAL4, UAS-GFP x UAS-*scro*-RNAi (TRiP)<br>Heatshock (37°C) for 10 mins at 24h ALH and 25°C until L3 wandering
Fig. 7H: Scro misexpression on Ey	Crtl: *insc*-GAL4>UAS- mCD8-GFP, tubGAL80^{ts} x w^{1118}<br>Scro OE: *insc*-GAL4>UAS- mCD8-GFP, tubGAL80^{ts} x UAS-Scro<br>18°C until hatching, 29°C until wandering L3
Fig. 7I: Hbn misexpression on *scro*	Crtl: *insc*-GAL4>UAS-mCD8-GFP, tubGAL80^{ts} x w^{1118}<br>Hbn OE: *insc*-GAL4>UAS-mCD8-GFP, tubGAL80^{ts} x UAS-Hbn<br>18°C until hatching, 29°C until wandering L3
Fig. S6E: Grh misexpression on Hbn	Crtl: *D*-GAL4>UAS-IVS-mCD8-RFP x w^{1118}<br>Grh OE: *D*-GAL4>UAS-IVS-mCD8-RFP x UAS-Grh
Fig. S6F: *grh* knockdown on Hbn	Crtl: *D*-GAL4>UAS-IVS-mCD8-RFP x w^{1118}<br>*grh* RNAi: *D*-GAL4>UAS-IVS-mCD8-RFP x UAS-*grh*-RNAi
Fig. S6L: *scro* knockdown on D	Crtl: *D*-GAL4>UAS-IVS-mCD8-RFP x UAS-RNAi-mCherry<br>*scro* RNAi: *D*-GAL4>UAS-IVS-mCD8-RFP x UAS-*scro*-RNAi (TRiP)
Fig. S6M: *scro* knockdown on Grh	Crtl: *D*-GAL4>UAS-IVS-mCD8-RFP x UAS-RNAi-mCherry<br>*scro* RNAi: *D*-GAL4>UAS-IVS-mCD8-RFP x UAS-*scro*-RNAi (TRiP)

Table S3, Related to Figs. 5-7 and S5-6.

List of genotypes used in experiments.
